# Abscisic acid plays a key role in the regulation of date palm fruit ripening

**DOI:** 10.3389/fpls.2022.1066142

**Published:** 2023-02-17

**Authors:** Saar Elbar, Yochai Maytal, Itzhak David, Mira Carmeli-Weissberg, Felix Shaya, Yaara Barnea-Danino, Amnon Bustan, Smadar Harpaz-Saad

**Affiliations:** ^1^ The Robert H. Smith Institute of Plant Sciences and Genetics in Agriculture, Hebrew University of Jerusalem, Rehovot, Israel; ^2^ Ramat-Negev Desert Agro-Research Centre, Halutza, Israel; ^3^ Department of Fruit Tree Sciences, The Volcani Center, Agricultural Research Organization, Rishon LeZion, Israel; ^4^ Southern Arava Research and Development, Hevel Eilot, Israel

**Keywords:** abscisic acid (ABA), date palm, ethylene, fruit development, fruit maturation, fruit ripening, Medjool, *Phoenix dactylifera* L.

## Abstract

The date palm (*Phoenix dactylifera* L.) fruit is of major importance for the nutrition of broad populations in the world’s desert strip; yet it is sorely understudied. Understanding the mechanism regulating date fruit development and ripening is essential to customise date crop to the climatic change, which elaborates yield losses due to often too early occurring wet season. This study aimed to uncover the mechanism regulating date fruit ripening. To that end, we followed the natural process of date fruit development and the effects of exogenous hormone application on fruit ripening in the elite cultivar ‘Medjool’. The results of the current study indicate that the onset of fruit ripening occurre once the seed had reached maximum dry weight. From this point, fruit pericarp endogenous abscisic acid (ABA) levels consistently increased until fruit harvest. The final stage in fruit ripening, the yellow-to-brown transition, was preceded by an arrest of xylem-mediated water transport into the fruit. Exogenous ABA application enhanced fruit ripening when applied just before the green-to-yellow fruit color transition. Repeated ABA applications hastened various fruit ripening processes, resulting in earlier fruit harvest. The data presented supports a pivotal role for ABA in the regulation of date fruit ripening.

## Highlights

The plant hormone abscisic acid (ABA) promotes date fruit ripening, as indicated by the enhanced change in fruit color from green to yellow and the enhanced rate of sugar accumulation.

## Introduction

*Phoenix dactylifera* L. (date palm) is a perennial dioecious monocot tree. The date palm thrives in semi-arid to arid regions under severe climatic conditions, usually considered as abiotic-stress for most plant species (Al-Mssallem et al., 2013). The date palm fruit is a staple food for millions of people in the Middle East, North Africa, Asia and America due to its high energy content. Moreover, it also holds potential health benefits as a result of the high nutrient content and bioactive compounds like flavonoids, tannins, and other phenolic compounds (Lobo et al., 2014; Marondedze et al., 2014). However, despite its immense nutritional, cultural, and economic significance in these regions, the date palm is an under-studied crop (Al-Khayri et al., 2015).

The date palm thrives in areas characterized by high temperatures and low humidity (Al-Mssallem et al., 2013). The prerequisites for date fruit ripening are high temperatures and no precipitations during the phase of fruit development (Lobo et al., 2014). Heat unit requirements for fruit ripening vary with cultivar and can range between 1100 to 2500 hours above 18°C (Lobo et al., 2014). High humidity during the flowering season or at later stages of fruit development results in various physiological disorders and, hence, also limits the area of date fruit commercial production (Yahia and Kader, 2011). In recent years, traditional date palm regions have been affected by global climate changes, which too often drive earlier monsoons that have detrimental consequences on fruit quality and marketability (Haris et al., 2019; Hussain et al., 2020). In parallel, the rapid expansion of commercial date palm orchards to suboptimal climate regions has challenged the completion of fruit development and ripening before the late-summer temperature decline, humidity upsurge, and early rain events, which result in fruit deterioration and significant yield loss (Awad, 2007). Facilitated fruit development, particularly ripening, may reduce fruit deterioration and prevent the significant yield losses caused by adverse weather events.

Traditionally, date fruit development is categorized into five distinct stages represented by immature creamy-white (Hababouk), immature green (Kimri), maturing yellow fruit (Khalal), soft brown (Rutab) and hard raisin-like (Tamr) fruits, respectively (**Supplementary Figures S1, S2**; Chao and Krueger, 2007). Following pollination, there is a 4-6-week phase of stagnation, during which no growth in fruit size or biomass can be detected (Hababouk). Toward the end of this stage, one ovule in each carpellate flower develops into a fruit, whereas the other two carpels degenerate (Slavković et al., 2016). Then the selected ovule grows through a combination of cell divisions and cell expansions until the fruit reaches its maximum size (Kimri). During this stage, there is the continuous increase in reducing sugars while moisture content is at a peak (up to 85%; Barreveld, 1993). Once the fruit reaches maximum size, the seed is fully mature (can germinate if dispersed) and ripening of the fruit pericarp begins (Khalal). This involves gradual chlorophyll degradation and carotenoids processing, leading to fruit color change from green to yellow alongside an increase in fruit sucrose concentrations (Barreveld, 1993; Bernstein, 2004). When the pericarp Brix value reaches about 40% (Ben-Zvi et al., 2017), fruit color changes from yellow to brown (Rutab), which typically starts at the distal tip and progressively continues towards the calyx end. During this phase, sucrose is converted to reducing sugars, the fruit softens and tannins precipitate, leading to astringency loss. Finally, the reduction in fruit water content yields a dry fruit with a sugar content of 70-80% (Tamr), a phenomenal level that has been documented so far only in date fruit (Barreveld, 1993; Lobo et al., 2014).

Fruit ripening is a complex process during which various metabolic pathways are orchestrated in a timely manner. These include chlorophyll breakdown, pigment synthesis, sugar accumulation, acid degradation, aroma-related volatile production and other processes (Peng et al., 2022; Wang et al., 2022). Traditionally, fruit ripening is classified as climacteric vs. non-climacteric. In climacteric fruit, ripening is characterized by a distinct peak in ethylene production followed by an abrupt peak in respiration. In such fruit, ethylene functions as a key regulator, coordinately activating a wide arsenal of processes leading to fruit ripening (Klee and Clark, 2010; Kou et al., 2021). In non-climacteric fruits, no significant increase in respiration or ethylene production is detected during the course of fruit ripening. Instead, accumulating data exploring the mechanism underlying the regulation of non-climacteric fruit ripening suggest a key role for the plant hormone abscisic acid (ABA; Wills and Ku, 2002; Jia et al., 2011; Zaharah et al., 2012; Ferrero et al., 2018; Karppinen et al., 2018; Gupta et al., 2022; Li et al., 2022). In certain cases, ethylene is involved in the regulation of specific aspects of non-climacteric fruit ripening but not in the coordinated induction of the entire process (Stewart and Wheaton, 1972; Katz et al., 2004; Li et al., 2016; Chen et al., 2018; Farcuh et al., 2018).

In dates, there is an ongoing debate about the mechanism that regulates fruit ripening. A number of studies suggest that date fruit ripening follows a climacteric pattern. These are based on a small peak in ethylene production at early stage of date fruit ripening reported for ‘Negros’ cultivar (Serrano et al., 2001), an increase in ethylene production during fruit ripening reported for ‘Zahdi’, ‘Derey’, ‘Sultani’ and ‘Hillawi’ cultivars (Abbas and Ibrahim, 1996), and additional reports demonstrating that ethylene application facilitates date fruit ripening pre- and post-harvest (Musa, 2001; Awad, 2007; Al-Qurashi and Awad, 2011; Al-Saif et al., 2017). However, other reports suggest that date fruit ripening follows a non-climacteric pattern, as the rate of ethylene production and respiration decreases during fruit ripening (Rouhani and Bassiri, 1976; Lobo et al., 2014), the level of proteins required for ethylene production decrease during fruit ripening (Marondedze et al., 2014) and exogenous ethylene application does not enhance date fruit ripening (Rouhani and Bassiri, 1977; Aljuburi et al., 2001). The information about the role of ABA in date fruit ripening is scarce. Awad (2007) and, more recently Shareef and Al-Khayri (2020), provided evidence associating an increase in endogenous ABA concentrations with fruit ripening in the cultivars ‘Helali’ and ‘Hillawy’ (Awad, 2007; Shareef and Al-Khayri, 2020). However, both studies focused on the stage of fruit color transition from yellow-to-brown (from ‘Khalal’ to ‘Rutab’) the final stage in fruit ripening; there have been no reports regarding the role of ABA during earlier stages of date fruit ripening. Here we aimed to investigate the mechanism regulating date fruit ripening and specifically, to study the role of ABA in this process.

## Results

### Fruit development and ripening of ‘Medjool’ date fruit

**Figure 1:**
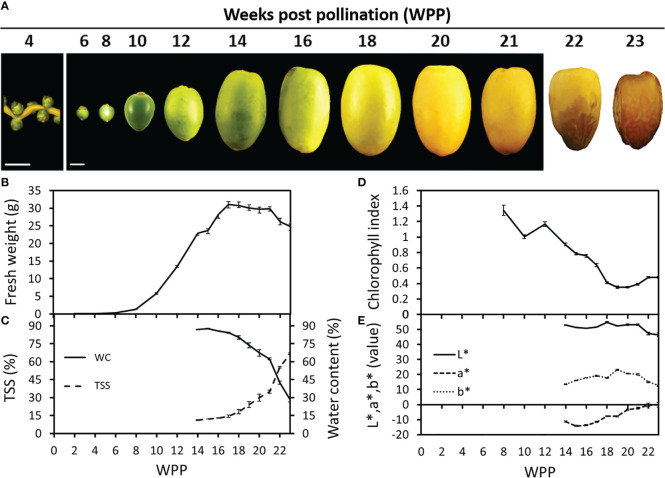
Date palm fruit development. Representative fruits were sampled and photographed along the course of fruit development (Grofit 2017). Numbers indicate weeks post-pollination **(**WPP; **A)**. Fruit development was characterized through quantification of **(B)** fruit fresh-weight, **(C)** total soluble solids (TSS; %) and water content (WC; %), **(D)** chlorophyll relative fluorescence and **(E)** L*a*b* color space parameters. Error bars indicate standard error. Scale bar: 1 cm. The number of fruits measured at each time point was **(B, D, E)** n=18 or **(C)** n=6.

The natural process of ‘Medjool’ date fruit development was monitored from pollination until harvest. During the first six weeks post-pollination (WPP), no substantial changes in fruit size or shape were observed (**Figure 1**). However, starting from 6 WPP, extensive fruit growth was detected, reaching maximum fresh weight at about 17 WPP (**Figures 1A, B** and **Supplementary Figures S3A, B**). Then at 22 WPP, following 4 weeks (17-21 WPP) during which no significant changes were observed, fruit fresh weight decreased until harvest (**Figure 1B** and **Supplementary Figure S3B**). The reduction in fruit weight during the last phase of fruit development, was in accordance with a sharp decrease in fruit water content (WC) detected from 21 WPP until harvest (**Figure 1C**). Sugar accumulation in the fruit, measured as the pericarp total soluble solids (TSS), ranged between 10% and 15% until week 16, after which a significant increase was detected, gradually rising to about 35% by 21 WPP, and then sharply shooting up to about 70% in the ready-to-harvest, fully ripe fruit (**Figure 1C**). The chlorophyll fluorescence index revealed a continuous reduction in fruit-exocarp chlorophyll levels, which began at 10-12 WPP (**Figure 1D** and **Supplementary Figure S3C**), reaching a minimum value of ~0.4 fluorescence index units (SFR_G) by 19 WPP, which paralleled the complete fruit exocarp color transition from green to yellow (**Figures 1A, D** and **Supplementary Figures S3A, C**). In addition, chromameter measurements of the fruit L*, a*, b* color space revealed a distinct decline in the green color, as indicated by the increase of a* between 17 WPP and 22 WPP, accompanied by an increase in the yellow color component indicated by b*, which peaked in 19 WPP, remained stable until 21 WPP and then gradually decreased. Color brightness (L*) remained stable until 21 WPP, and then declined in association with fruit exocarp color transition from yellow to brown (**Figures 1A, E**). Similar, but much stronger trends were also observed in the following year (**Supplementary Figures S3A, D**).

**Figure 2:**
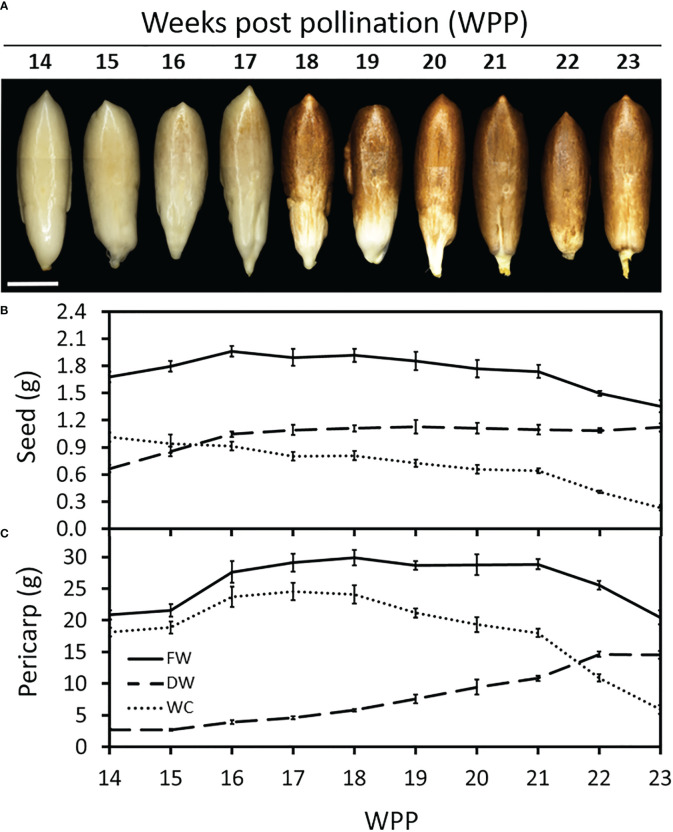
Date palm seed and fruit development. Representative fruits were sampled and seeds were separated from the pericarp tissue (Grofit 2017). **(A)** Seeds were photographed at different stages in fruit development as indicated by number of weeks post pollination (WPP). Dry weight (DW) and water content (WC) were quantified for both **(B)** seed and **(C)** pericarp. Note the substantial reduction in WC, in both seed and fruit pericarp, starting from 21 WPP. Error bars indicate standard error. Scale bar: 1 cm. The number of fruit measured at each time point was n=6.

Monitoring seed development along the course of ‘Medjool’ date fruit development, provided insight into the interrelationship between these two organs. Seed color gradually turned brown from 17 WPP until 21 WPP, when it was completely brown (**Figure 2A**). Seed growth, as measured by dry weight, continued until 16 WPP, when the seed reached its final biomass (**Figure 2B**). Seed WC decreased consistently from 13 WPP until 21 WPP and then sharply declined until harvest (**Figure 2B**). In contrast to the seed, pericarp growth in dry weight recommenced at 15 WPP and gradually increased until 22 WPP, while its WC increased until 16 WPP, remained stable for about two weeks, declined slowly until 21 WPP, and then dropped sharply until fruit harvest (**Figure 2C**). Note that seed biomass accumulation was completed by 16 WPP, shortly before the fruit reached its maximum size, and prior to fruit color change from green to yellow that occurred at 17-18 WPP (**Figure 1** and **Supplementary Figure S3**).

Altogether, the data accumulated over the two seasons, imply two major regulatory junctures. The first, as the seed biomass reaches the maximum weight value, about 1-2 weeks before the pericarp tissues reach the maximum fresh weight (**Figure 2**). This stage seems to coincide with the induction of fruit ripening, associated with fruit color transition from green-to-yellow. The second, is just before the final stage in date fruit development, when a sharp reduction in fruit water content is coupled with a sharp increase in pericarp sugar levels. These changes coincide with the final fruit color transition from yellow-to-brown, as the fruit becomes ready to harvest.

### Water transport to the fruit pericarp is interrupted prior to the yellow-to-brown transition

**Figure 3:**
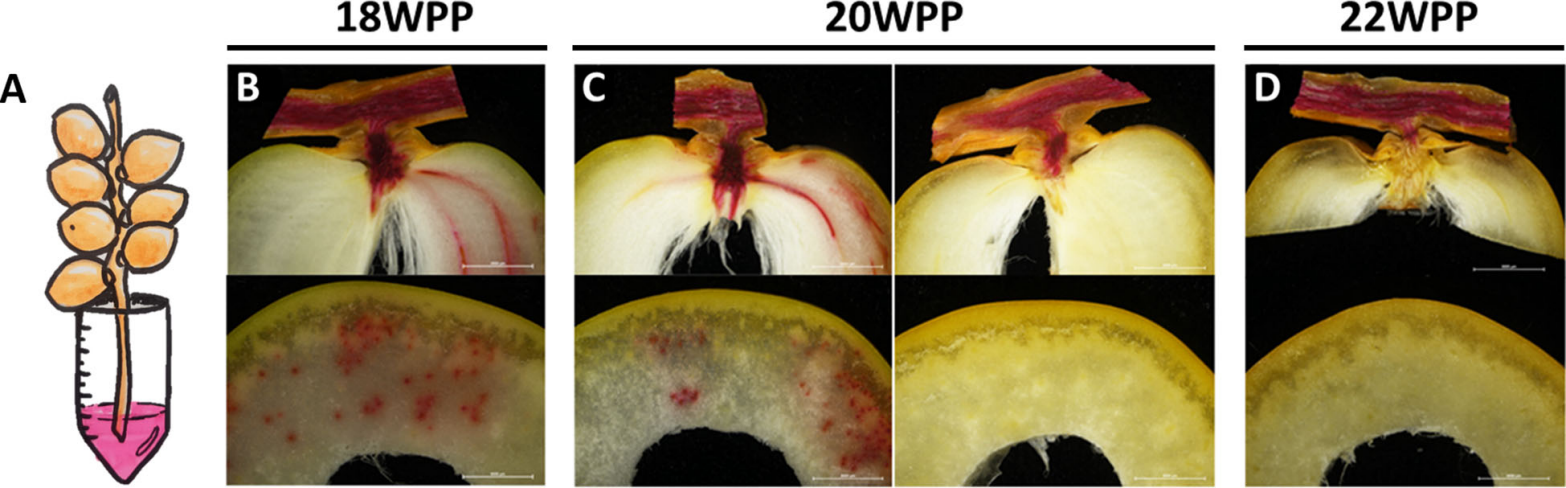
Vascular water flux arrest from the strand to the fruit occurs as part of date fruit development. Representative fruit-bearing strands were sampled along the course of fruit development (Grofit 2017) and movement of the dye Safranin O to the fruit was monitored **(A)** at different stages in fruit development using cross and longitudinal sections prepared **(B)** 18, **(C)** 20, and **(D)** 22 weeks post-pollination (WPP). Scale bar: 0.5 cm. Number of sampled strands at each time-point was n=3, each bearing 6-15 dates.

In order to understand the processes regulating the dynamics of fruit water status, the xylem flow was followed at different stages in fruit development using the water-soluble, xylem-transportable dye safranin-O. Fruit-carrying strands (**Supplementary Figure S1**) were detached at different stages along the course of fruit development, placed in a safranin O solution, and then examined for dye appearance in both the strand vascular system and the fruit pericarp (**Figure 3** and **Supplementary Figures S4**, **S5**). Soon after pollination, the dye was observed in all female flowers examined, and reached either all three independent ovaries of the female flower or only one of the three (**Supplementary Figure S4**). During the date fruitlet abscission period (5 WPP), the staining method easily discriminated between fruitlets prone to abscise and those due to persist (**Supplementary Figure S5**). In fruitlets that were prone to abscise, which detached from the strand in response to very slight mechanical caress, the dye streaming that was detected in the strand’s vascular system avoided the fruitlet tissues. In contrast, no interruption of the dye stream was observed in the persisting, strongly-attached fruitlets. From 6 WPP to 18 WPP, xylem transport to the fruit was not interrupted and the dye was observed in the vascular system of both the strand and the fruit pericarp (**Figure 3** and **Supplementary Figure S6**). Yet, at 20 WPP, while the strand’s vascular system was stained in all samples, the pericarp was stained only in half of the fruits examined (**Figure 3** and **Supplementary Figure S6**). Later on, from 22 WPP until harvest, the dye was observed only in the strand and pedicel vascular system but not in the pericarp (**Figure 3** and **Supplementary Figure S6**). Interestingly, the timing of the vascular continuity disruption, at 20-21 WPP, coincided with the sharp decline in fruit and seed WC (**Figures 1**, **2** and **Supplementary Figure S3**) and the sharp increase in pericarp sugar content (**Figure 1C**) and dry weight (**Figure 2C**). To summarize, these results suggest that prior to the yellow-to-brown fruit color change, xylem-mediated water transport from the strand to the fruit is interrupted as part of the natural process of ‘Medjool’ date fruit development.

### Endogenous ABA levels increase during date palm fruit development

**Figure 4:**
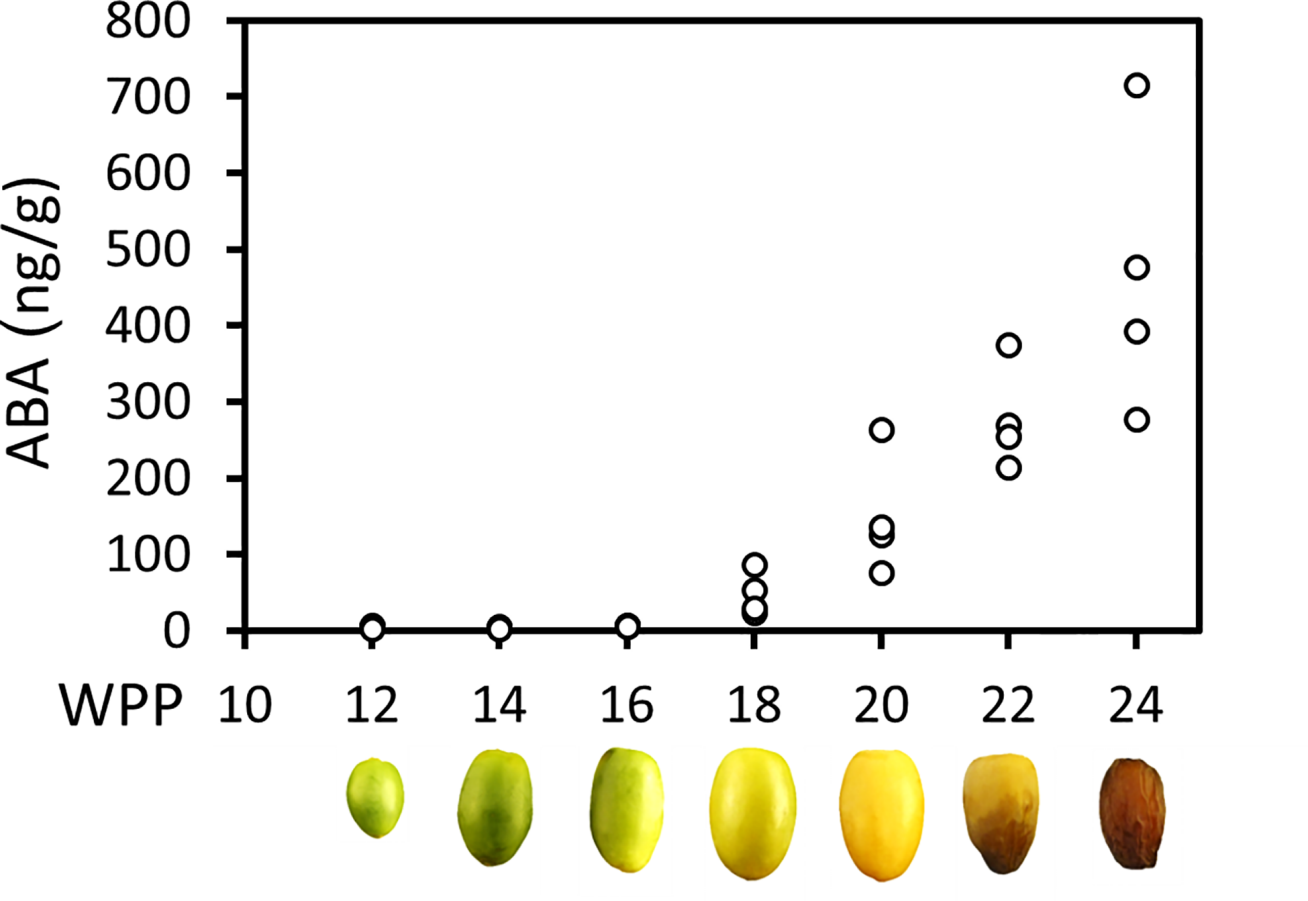
Endogenous ABA level increase throughout date palm fruit ripening. Endogenous ABA levels in representative fruits were quantified along the course of fruit development (Grofit 2017), using LC-MS. At each time point, four independent fruits served as biological replicates. ABA content was normalized to fresh weight.

Endogenous ABA in the fruit pericarp was undetectable until 16 WPP. Significant endogenous ABA levels were detected starting from 18 WPP at the onset of fruit ripening, and continuously increased until fruit harvest (**Figure 4**). The rise in pericarp ABA in parallel to the process of fruit maturation suggests a regulatory role for this plant hormone in the ripening process of ‘Medjool’ date fruit.

### Exogenous ABA application facilitates fruit ripening

**Figure 5:**
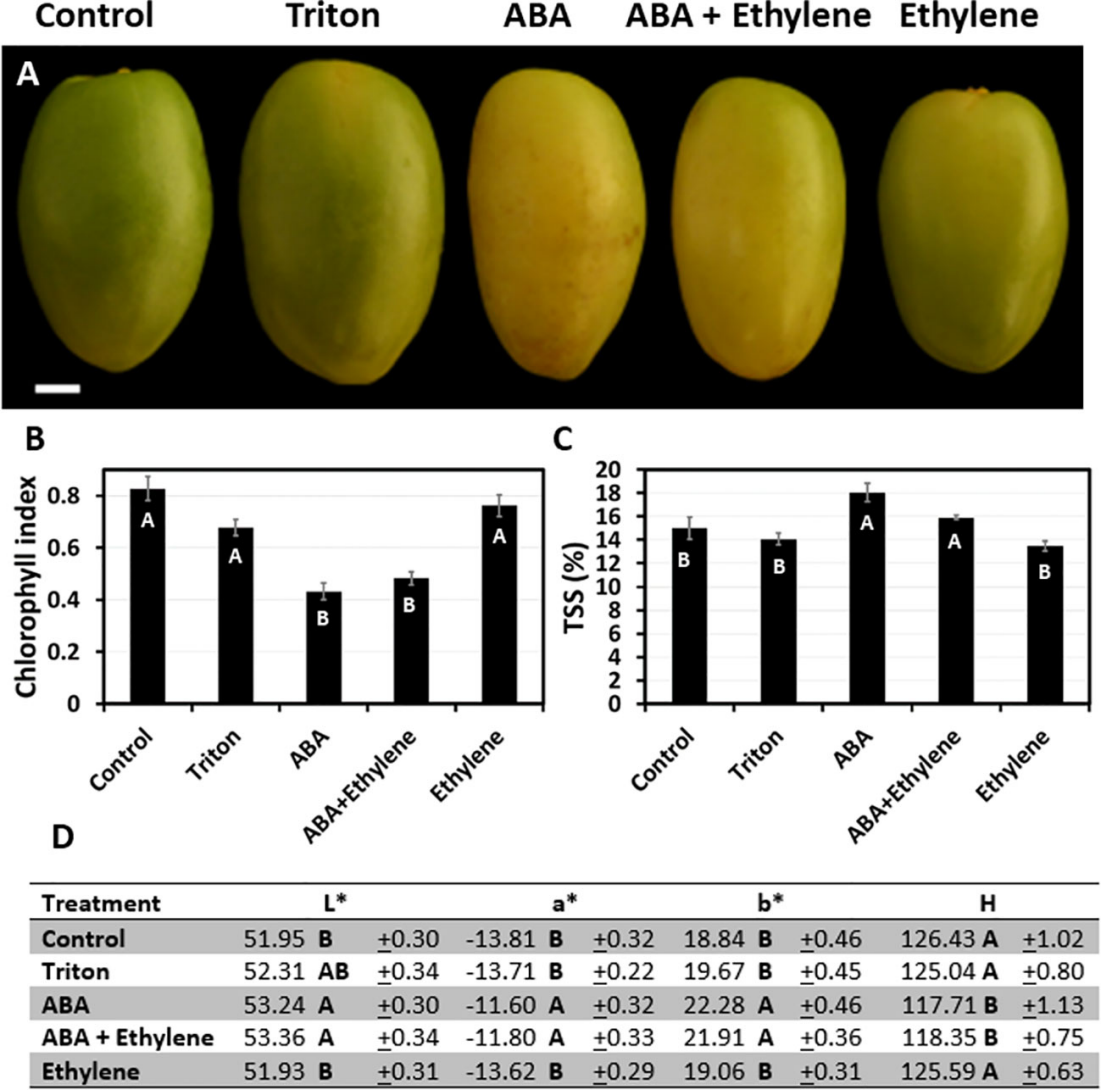
The effect of exogenous ABA and ethylene treatment applied at 16 WPP, on date fruit development. Date fruits were treated with a single treatment of ABA (ProTone™), ethylene (Ethrel^®^) or a combination of both, at 16 WPP on the tree (Grofit 2017). **(A)** Representative fruits from each treatment group were sampled and photographed one week after the treatment. Fruit ripening was characterized through quantification of **(B)** chlorophyll relative fluorescence, **(C)** total soluble solids (TSS; %), and **(D)** color index. Error bars and “±” indicate standard error. Letters represent Tukey-Kramer multiple comparison test **(B**; p-value < 0.0001, C, D; p-value < 0.05**)**. Scale bar: 1 cm. Number of fruits measured per treatment at each time-point: **(B, D)** n=12, or **(C)** n=6.

To better understand the role of ABA, ethylene, or a combination of the two, in date fruit ripening, individual fruiting strands were treated in the orchard with the selected hormone at 14, 16, or 20 WPP. When treated at 14 WPP, prior to a visible green-to-yellow change in fruit exocarp color, the combined ABA + ethylene treatment triggered the most prominent affect, as it facilitated exocarp color transition from green to yellow and led to a transient increase in TSS levels (**Supplementary Figures S7**, **S8**). When applied at 16 WPP, both ABA and the combined ABA + ethylene had similar effect in initiating fruit maturation, manifested by significant green-to-yellow color changes, and an increase in TSS levels as compared to ethylene and control treatments (**Figure 5** and **Supplementary Figure S9**). Nevertheless, the impact of the exogenously applied ABA or ABA + ethylene at 14 and 16 WPP was transient, lasting for only 1-2 weeks after treatment (**Supplementary Figures S8**, **S9**). When applied at 20 WPP, none of the hormone treatments affect fruit development, as most of the fruit had already launched their natural maturation processes (**Supplementary Figure S10**).

**Figure 6:**
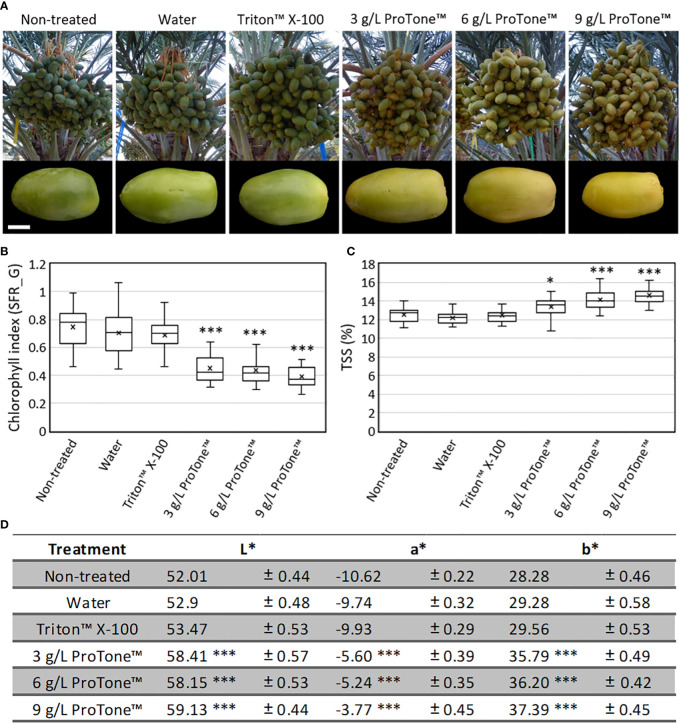
Effects of exogenous ABA treatment on date fruit maturation. Whole date fruit clusters were treated every three days (four treatments in total) with 3, 6 or 9 grL^-1^ ProTone™ (ABA), starting at 17 WPP, on the tree (Grofit 2020; **A**; upper panel). Representative fruits per treatment were sampled and photographed one week after the last treatment **(A**; lower panel**)**. Fruit ripening was characterized through quantification of **(B)** chlorophyll relative fluorescence, **(C)** total soluble solids **(**TSS; **D)** and the L*a*b* color space parameters. Error bars and “±” indicate standard error. All data were analyzed using Tukey-Kramer multiple comparison test **(B, C, D)**; * indicates p-value < 0.5; *** indicates p-value < 0.0001. The number of fruits measured per treatment at each time point was n=30.

Repetitive exposure (4 applications over 13 days) of whole clusters to the ABA treatment, starting on 17 WPP, when the green fruit is at its maximum size and before the green-to-yellow transition, led to yellowing of all fruits, while all control fruits remained green (**Figures 6A, B, D**). In addition, TSS levels of all ABA-treated fruits were significantly higher compared to those of control fruits (**Figure 6C**).

**Figure 7:**
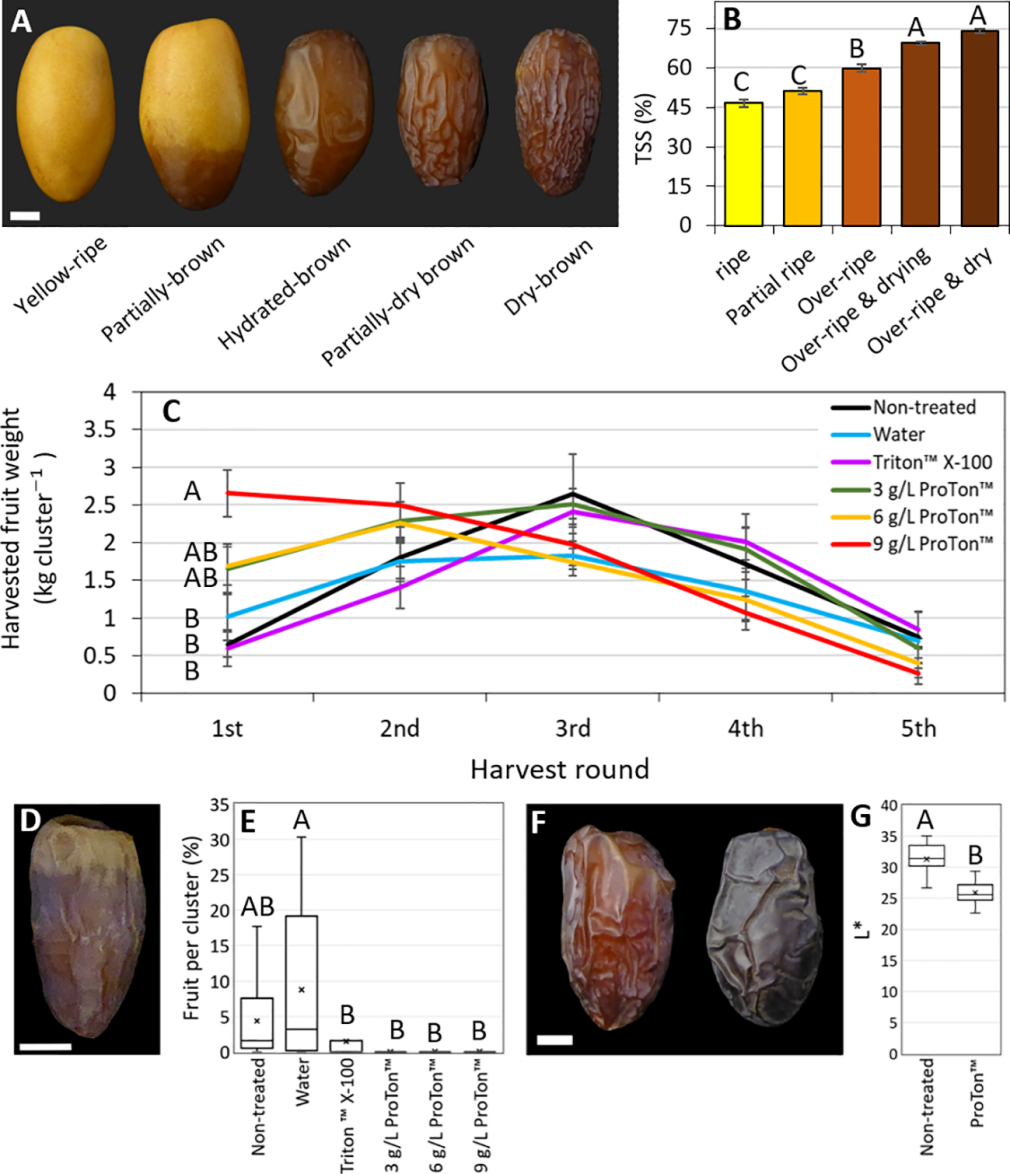
Pre-harvest ABA treatments promote fruit harvest and result in darker fruit. In the season of 2020, whole date fruit clusters were treated pre-harvest every three days (four treatments in total) with ABA (ProTone™; 3, 6 or 9 g/L), and Triton™ X-100, starting from 17 WPP. At harvest, five ripening classes were defined by **(A)** appearance and **(B)** total soluble solids % (TSS). **(C)** The weight of the fruit harvested from the treated clusters at each of the five harvest rounds (one round per week), is shown. **(D)** Typically, the first harvested fruits of this very arid habitat, are not marketable since they are too dry and do not ripen properly. **(E)** The percentage of such nonmarketable fruits in the first harvest round of the season was calculated. **(F)** Fruits treated with ABA appeared darker then the non-treated fruits. **(G)** Representative fruits per treatment were sampled 77 days after treatment, photographed, and measured for L* parameter of the L*a*b* color space. Error bars indicate standard error **(B, C)**. X mark indicates mean **(E, G)**. Data was analyzed using Tukey-Kramer multiple comparison test **(B**; p-value < 0.0005, C and E; p-value < 0.05, G; p-value < 0.0001**)**. The number of fruits measured for each class was n=6 **(B)**. The number of clusters harvested per treatment at each time-point was n=10 **(C, E)**. The number of fruits measured for each treatment was n=30 **(G)**. Scale bar: 1 cm **(A, D, F)**.

Harvest of this experiment, Grofit 2020, began at 25 WPP, and was done in 5 rounds. Total fruit yield was in the range of 6.1-8.5 kg cluster^-1^, with some tendency for higher yields in the ABA-treated clusters (**Supplementary Figure S11A**). While the number of fruits per cluster substantially fluctuated across treatments (**Supplementary Figure S11B**), mean fruit weight at harvest was significantly greater in samples treated with the higher ABA levels (6 and 9 g L^-1^ ProTone™), as compared to control and water-applied fruit (26.8 g fruit^-1^ vs. ~22.6 g fruit^-1^ and 22.2 g fruit^-1^ for non-treated and water-treated control; **Supplementary Figure S11C**). Interestingly, fruit treated with a surfactant (Triton-X100) or with the lower ABA level (3 g L^-1^ ProTone™) displayed intermediate values regarding fruit weights (24.5 g and 25.4 g, respectively). When compared to controls, a much greater proportions of the ABA-treated fruit yield was harvestable in the first round (20-32%, compared to 8-15% of the total yield, respectively). On treatment with one of the two higher ABA concentrations tested, more than 60% of the fruit were harvested in the 1st and 2nd rounds, while in the non-treated and surfactant-treated control, 60-65% of the fruit were harvested in the 3^rd^ and 4^th^ rounds. Harvest from clusters treated with water, surfactant or a low ABA dose distributed quite impartially, with about 80% of the fruit collected in rounds 2 to 4. In the fifth round of harvest, only residues were left (up to 5% of the total yield), with no significant differences between treatment-groups (**Figure 7C**). Thus, repetitive ABA treatments accelerate date fruit ripening and precede fruit harvest.

In addition, it is noteworthy that the fraction of yellow fruit that easily abscised at harvest was much greater among the clusters exposed to repetitive ABA treatments, reaching 20–30% of the fruit, compared to about 3% in the controls (**Supplementary Figure S12**). Moreover, the fraction of partially brown fruit was higher among the control groups (14–18%), as compare to ABA-applied fruits (10-11%). Especially notable was the scarcity (less than 1%), of partially dry and dry brown fruit fractions (hydrated and dry ‘Tamr’) and the absolute dominance of hydrated brown fruit (‘Rutab’) in the ABA-treated samples, as compared to 8-15% in the controls (**Supplementary Figure S12**), suggesting that ABA may also affect the last stage of fruit dehydration. Subsequently, visual and spectral differences in fruit appearance were apparent; the surface of ABA-applied fruits was considerably smoother and darker compared to control fruit (**Figures 7F, G**). Last, repetitive ABA treatments, like the surfactant alone, reduced the fraction of prematurely-dry fruit in comparison to the non-treated or water-treated controls (<2% vs. 10-20%; **Figures 7D, E** and **Supplementary Figure S12**). Taken together, repetitive ABA treatments can accelerate date fruit ripening.

## Discussion

The current study, aimed at characterizing the role of ABA in date fruit ripening, identified gradual increase in endogenous ABA levels during natural fruit ripening. One-time exogenous ABA application accelerated the process in a transient manner, while repetitive ABA treatments enhanced fruit ripening and resulted in early fruit harvest. Interestingly, ethylene treatment did not promote fruit ripening, unless applied at very early stages in fruit development together with ABA, suggesting a possible role for ethylene in the very early stages of ripening induction. Altogether, the presented data support a key role for ABA in the regulation of the timing and progress of the complex process of date fruit ripening.

### Endogenous ABA in date fruit ripening

While the role of ABA in the regulation of fleshy fruit ripening is well established, in dates this kind of information is scarce. In the present study, the endogenous ABA levels in date fruit pericarp are shown to rise at the point of fruit green-to-yellow transition, at about 18 WPP, and to consistently increase throughout fruit ripening (**Figure 4**). A similar pattern of constant gradual increase in ABA levels, was reported also for fruit ripening in oil palm, strawberry, litchi, sweet cheery and other fruit species (Wang et al., 2007; Tranbarger et al., 2011; Symons et al., 2012; Yeap et al., 2017; Li et al., 2022; Vignati et al., 2022). An alternative pattern, in which a transient increase in ABA levels at very early stages of fruit maturation triggers the onset of fruit ripening-related processes, was identified in various fleshy fruit species, including blueberries, grapes, mango, tomato, cucumber, persimmon, and others (Wheeler et al., 2009; Zaharah et al., 2012; Karppinen et al., 2013; Leng et al., 2014). In these species, an ethylene peak often accompanies the ABA peak suggesting that both hormones participate in triggering fruit ripening. The pattern of a continuous buildup of endogenous ABA levels, as shown here for date fruit, suggests a relentless and likely multi-layered rather than a transient role for ABA in the regulation of fruit maturation and ripening. Additionally, in oil palm, three ABA-responsive transcription factors were shown to be activated by WRINKELD 1, a master regulator that activates oil synthesis-associated genes, as part of the process of oil palm fruit ripening (Yeap et al., 2017). Altogether, the pattern of endogenous-ABA accumulation during the natural process of date fruit development suggests a complex role for ABA in the regulation of date fruit ripening.

### Exogenous ABA applications enhance date fruit ripening

To further characterize the role of ABA in triggering fruit maturation, date palm samples were exposed to single or repeated exogenous ABA application. The stage at which the hormones were applied had an immense impact on the results obtained. When hormone treatment was applied at 14 WPP, a synergetic effect of the combined ABA and ethylene treatment was observed on fruit exocarp color change (green-to-yellow; **Supplementary Figure S7**), supporting a role for both hormones in early induction of date fruit ripening. In contrast, when applied later in fruit development (16 and 20 WPP), this synergistic hormonal effect was not detected (**Supplementary Figures S9**, **S10**). Similar synergy between ABA and ethylene was identified in several studies on fleshy fruit ripening in either climacteric or non-climacteric fruits (Zhang, 2014). In litchi, defined as a non-climacteric fruit, ABA did not enhance fruit maturation when applied alone; however, when combined with ethylene, it enhanced both chlorophyll degradation and anthocyanin accumulation, supportive of enhanced fruit ripening (Wang et al., 2007). A reciprocity/cross-talk between ethylene and ABA was also reported for grape vine, tomato, fig and persimmon (Zhang et al., 2009; Sun et al., 2010; Zhao et al., 2012; Lama et al., 2019). In date palms, a small peak of ethylene production was previously reported at about seed maturation (Serrano et al., 2001); yet, so far, this peak was not assigned function in the regulation of fruit ripening. The possibility that a transient peak in ethylene production emerges from the seed-triggering ABA production in the fruit pericarp and thereby induces the process of fruit ripening, requires further research.

As fruit development proceeded, ABA alone was sufficient to enhance date fruit ripening, as indicated by both chlorophyll degradation and sugar accumulation (**Figures 5**, **6** and **Supplementary Figure S8**). Similar results were reported for other crops. For instance, in mango, which is a climacteric fruit, exogenous ABA treatment promoted fruit coloration and softening compared to control fruit, while an opposite effect was observed when ABA synthesis was inhibited by nordihydroguaiaretic acid (NDGA) treatment (Zaharah et al., 2012). In this context, it is noteworthy that in our experimental setting neither Fluridon, inhibiting the carotenoid pathway, nor NDGA inhibited date fruit ripening (data not shown). In peach, also a climacteric fruit, exogenous ABA application facilitated sugar accumulation (Kobashi et al., 2001). Notably, in the current study, single ABA application displayed a short-term effect on the ripening process and hence, multiple applications were required to achieve durable enhancement of fruit ripening and advance fruit harvest. An exception was noted for date palm samples treated at 20 WPP, which showed no measureable effect of ABA on fruit ripening (**Supplementary Figure S10**), suggesting that ABA is sufficient to facilitate date fruit ripening when applied at the appropriate time during fruit development. Taken together, before practical ABA application is considered, it will be essential to determine the optimal treatment schedule and to improve methodologies aiming to enhance the penetration of the active substance into the fruit and to ensure sufficient duration of its impact.

### The role of ethylene in date fruit ripening

Traditionally, fruit ripening is classified as climacteric or non-climacteric. Climacteric fruit ripening is characterized by a distinct ethylene peak, which is followed by an abrupt peak in respiration. In climacteric fruit ripening, ethylene functions as a key regulator, coordinately activating a wide arsenal of processes leading to fruit ripening. Hence, ethylene application efficiently induces fruit ripening in climacteric fruits (Kou et al., 2021; Peng et al., 2022). In non-climacteric fruit, ethylene may induce specific aspects of fruit ripening but not the entire coordinated process (Fan et al., 2022). In dates, there is an ongoing debate about the mechanism regulating fruit ripening. While a number of studies report an increase in ethylene production during fruit ripening (Abbas and Ibrahim, 1996; Serrano et al., 2001), others report a decrease in ethylene production and fruit respiration during fruit ripening (Rouhani and Bassiri, 1976; Feygenberg et al., 2010; Lobo et al., 2014). Moreover, a number of studies report that ethylene application facilitates date fruit ripening pre- and post-harvest (Musa, 2001; Awad, 2007; Al-Qurashi and Awad, 2011; Al-Saif et al., 2017), while others report that exogenous ethylene application does not facilitate fruit ripening (Rouhani and Bassiri, 1977; Aljuburi et al., 2001). Even though ethylene production was not measured as part of the current study, exogenous ethylene application at different stages in fruit development (14, 16, 20 WPP) was not sufficient to induce or accelerate date fruit ripening at any of the stages examined (**Figure 5** and **Supplementary Figures S7**-**S10**). Combined with the pronounced effect detected for ABA in accelerating date fruit ripening, the findings of the current research suggest that fruit ripening in date palm cv. ‘Medjool’ follows a non-climacteric pattern.

### The final stage in fruit ripening is associated with disruption of xylem-mediated water transport

Due to the harsh arid environment in which dates grow, water dynamics during date fruit development and ripening has been the focus of many studies (Aldrich et al., 1946; Rygg, 1946; Gribaa et al., 2013; Garcia-Maquilon et al., 2021). The modifications in date fruit water status throughout its development are unique; from the initial phase of fruit growth, water levels rise constantly until they reach very sharp maxima as the fruit reaches its final size. From that point and until harvest, fruit WC continuously declines (Bernstein, 2004; Chao and Krueger, 2007; Lobo et al., 2014), in parallel to the gradual increase in fruit pericarp ABA levels (**Figures 1**, **2**, **4**). The relationship between water stress and ABA levels is well studied in a number of fleshy fruit crops (González and Iusem, 2014). A key component identified in that relationship is the water stress-inducible ABA STRESS RIPENING (ASR) transcription factor, which has been well-linked to ripening initiation in crops such as grape, tomato, strawberry, banana and others (Çakir et al., 2003; Gupta et al., 2006; Golan et al., 2014; Jia et al., 2016; Zhao et al., 2021). ABA also regulates the function of various aquaporins, which mediate water transport between cells and tissues (Fang et al., 2019), including in developing fruit (Breia et al., 2020). ABA has been also recently implicated in the regulation of the fruit cuticle deposition in orange (Wang et al., 2016) and in cucumber (Wang et al., 2015) which adds additional aspect to the way it might regulate fruit water status.

After water loss initiation, the next interesting point in water kinetics is the rapid decline in fruit WC just before fruit color transition from yellow to brown. While, xylem-mediated water flux into the fruit was maintained until 20 WPP, at 21-22 WPP, it stopped abruptly just before to the yellow-to-brown transition (**Figure 3** and **Supplementary Figures S4**, **S5**). Modifications in the vascular tissue during fruit development and ripening were recently identified and thoroughly investigated in grapes. At véraison, the onset of fruit ripening, water flux into the fruit gradually shifts from the xylem to the phloem (Bondada et al., 2005; Choat et al., 2009; Keller et al., 2015). A more recent study suggested a scenario, in which a discharge of surplus phloem water *via* berry transpiration and/or xylem backflow to the pedicel, may be necessary to facilitate natural grape ripening as well as sugar accumulation (Zhang and Keller, 2017). Interestingly, a partial fracture of the cluster’s stem, which considerably decreased the water flow to the fruit, was shown to accelerate the ripening of ‘Medjool’ date fruit, when executed at the yellow-to-brown transition, also known as the ‘Khalal-to-Rutab’ shift, suggesting that severe water deficit can trigger the final stage of ripening in mature dates (Bernstein, 2004). Considering the above, we hypothesize that water loss starting at ‘color break’ might elicit a stress signal that induces a series of events facilitating fruit ripening, specifically the last phase of the yellow-to-brown transition.

### Is ABA a multi-tasking hormone?

Soluble sugar levels in ripe fleshy fruits display large variations across species (Coombe, 1976), with very low °Brix (~ 4) in tomato (Wills and Ku, 2002), moderate (~11°Brix) in peach (Bregoli et al., 2002), and high (~24°Brix) in wine grapes (Bregoli et al., 2002; Wills and Ku, 2002; Bondada et al., 2017). Date palm fruit are unique in this sense, reaching 44–50° Brix at full maturity, as the fruit turns from yellow-to-brown (**Figure 1C**), and 70 – 80° Brix at the ripe-dry ready-to-harvest stage, also known as ‘Tamr’ (Chao and Krueger, 2007; Lobo et al., 2014). Such high °Brix levels are found only in grape raisins (Aung et al., 2002). In both species, the upsurge in fruit sugar concentration is strongly correlated with a significant decline in the fruit WC (**Figure 1C**; Aung et al., 2002). Within the well-established pivotal and comprehensive role of ABA in the regulation of non-climacteric fruit ripening (Pilati et al., 2017; Forlani et al., 2019; Fuentes et al., 2019; Bai et al., 2021), of particular interest is its capacity to integrate changes in fruit sugar metabolism and water status as part of fruit maturation and ripening. Solid evidence suggest a regulatory role for sucrose, in coordination with ABA and other hormones, in the regulation of fruit ripening in grape, strawberry, tomato, apple and peach (Falchi et al., 2013; Jia et al., 2016; Jia et al., 2017; Ma et al., 2017; Olivares et al., 2017). In non-climacteric fruit, some evidence suggest a regulatory role for sucrose transporters (*SUT* genes), through which sugars and ABA interact to induce and govern sucrose accumulation and fruit ripening (Wang et al., 2013). Additionally, *ASR* gene expression was upregulated by both sucrose and ABA and even more by their combination, which subsequently promoted fruit ripening, whereas RNA interference delayed it (Jia et al., 2016). Recently, a study focusing on elucidating ABA signaling pathway in dates identified four *PYRABACTIN-LIKE 8* (*PYL8*) genes, among the 12 genes encoding for the ABA receptor that are expressed during date fruit development. Among these, *PYL8-LIKE 27* was the most highly expressed at the yellow-to-brown transition (**Supplementary Figure S2**; Garcia-Maquilon et al., 2021). The identity of the signal that regulates the timing of the natural increase in fruit pericarp ABA levels and how it is related to the onset of fruit ripening remains to be determined. Various hypotheses can be raised; i) a naturally induced water loss triggers a continuous stress signal, which leads to ABA production and consequently facilitates date fruit maturation and ripening, ii) cross-talk with other hormones that affect ABA metabolism, and last, iii) sucrose levels may regulate ABA accumulation.

### Practical implementations

The observations presented here may have significant practical implications. The date palm industry in Pakistan and India suffers from heavy summer monsoons occurring just before fruit ripening (Adel et al., 2015; Baidiyavadra et al., 2019). In northern Africa, early autumn rains and suboptimal temperature often overlap with the date ripening season (Musa, 2001; Awad, 2007; Lobo et al., 2014; Al-Khayri et al., 2015). In both cases, significant yield proportions are lost. Substantial variability in fruit ripening within a cluster, tree, and orchard cause considerable losses due to unripe or overripe unmarketable fruit. Within-cluster variability also necessitates many rounds of harvest, which is costly and limits date tree orchard size (Musa, 2001; Awad, 2007; Lobo et al., 2014; Al-Khayri et al., 2015). The ability to hasten date fruit development and regulate the timing of fruit harvest may facilitate expansion of date fruit orchards and cultivation area to habitats with sub-optimal growth conditions.

## Conclusions

Quite similar to véraison in grapes, seed maturation in date palm apprises the onset of fruit maturation and ripening. Endogenous ABA levels in date fruit pericarp gradually rise from the green-to-yellow transition, which is equivalent to véraison in grapes, until harvest. Exogenous ABA applications directly associate this hormone with fruit ripening, opening opportunities for practical influence on fruit harvesting time and fruit quality at harvest. Based on the data presented in the current study, we hypothesize that fruit ripening in ‘Medjool’ date palm follows a non-climacteric pattern and that ABA functions as a central regulator of this process. Further research is required in order to learn about the role of additional hormones in the regulation of date fruit ripening. It will be interesting to determine what regulates the timing and level of ABA accumulation in the fruit pericarp, how it affects the onset of fruit ripening and how it regulates downstream metabolic pathways induced/suppressed as part of the complex process of date fruit ripening.

## Materials and methods

### Plant material

All experiments were conducted on date trees of the cv. ‘Medjool’ at Grofit orchard, located in the Rift Valley at South Arava district (29° 56′ 27.02″ N, 35° 3′ 52.61″ E, 118 m above sea level), Israel. The experiments were conducted over three growing seasons (2017, 2018 and 2020), on 6-year-old tissue-culture-generated trees, starting from the second year of commercial harvesting. The soil was sandy, composed of 95% sand and 5% silt. In 2017, pollination was carried out with diluted pollen (25% pollen, 75% talc powder) for fruit load control. In 2018 and 2020, the pollen dilution was different (20% pollen, 80% talc powder). The pollen used in the current study was a mix of random pollen from different male date trees located in Grofit and the neighboring orchards. In all years, female inflorescences were trimmed as a fruit load management tool.

### Monitoring of fruit development and ripening over the growing season

Seasonal observations and monitoring of fruit development were carried out from pollination to the beginning of commercial harvest during the years of 2017 and 2018. All time points are presented as weeks post-pollination (WPP). The seasonal observation of date fruit development was conducted in a randomized block design, with 6 single-tree blocks. Four clusters per tree were chosen at anthesis and used for strands and fruit sampling. Eventually, each cluster carried approximately 200 fruits.

### Pre-harvest single-dose treatment with plant hormones

During the season of 2017, the hormone treatment experiment was laid out in randomized block design. Six trees were chosen at anthesis. One cluster per tree (six trees in total) was used, in which 3-5 strands (with a minimum of 18 fruit per treatment) were used for each treatment. For hormone treatments, 3-5 strands were dipped in plastic bags filled with hormone solution for 15 s, while avoiding exposure of other parts of the tree to the solution. The treatments included untreated control, water control, surfactant control - 0.1% Triton X-100 (Sigma-Aldrich), 6 g L^-1^ (containing 20% S-ABA as the active component) ProTone™ SG (Valent BioSciences) + Triton X-100 (0.1%), 2.1 ml L^-1^ Ethrel™ (Bayer) + Triton X-100 (0.1%) and a combined treatment of 6 g L^-1^ ProTone™ + 2.1 ml L^-1^ Ethrel™ + Triton X-100 (0.1%).

### Pre-harvest multiple-dose treatment with plant hormones

The 2020 experiments testing pre-harvest exogenous ABA application were extended to whole clusters. Comparable trees in the Grofit orchard were selected. Six uniform clusters from each tree (pollinated in the same week, and each carrying 300 fruits, on average) were chosen, and each one was designated to a different treatment. Thus, the experiment was performed in a randomized block design with 10 trees (each one is considered a block) and six treatments per tree. The treatments included untreated control, water control, 0.1% Triton X-100 (Sigma-Aldrich), or 3, 6, or 9 g L^-1^ (containing 20% S-ABA as the active ingredient) ProTone™ SG (Valent BioSciences) + Triton X-100 (0.1%). During application, each cluster was insulated using a plastic conus, to avoid undesired spray to neighboring treated clusters. Clusters were sprayed to drainage to ensure full coverage. Treatments were applied at dawn, at the daily peak of relative humidity and minimum wind, to maximize retention of the spray on the fruit surface. Applications were repeated 4 times, starting in 17 WPP, once every 3 days. One week after the first application, 3 representative fruits of each cluster were sampled and used for color and Brix determinations. Harvest was carried out in 5 rounds (once a week), upon occurrence of earliest ripening symptoms (one week before the start of commercial harvest in the plot). Each cluster was carefully shaken, and the fallen fruit were collected from its net-bag. All fruit from each cluster were counted and sorted according to their ripening stage, as follows: premature dehydration (PMD); yellow-ripe (‘Khalal’); partially-brown; hydrated-brown (‘Rutab’); partially-dry brown (hydrated ‘Tamr’); and, dry brown (dry ‘Tamr’; **Figure 7A** and **Supplementary Figure S12**). This classification was supported by fruit TSS, which steadily increased with the rise in ripening stage (**Figure 7B**). All fruits were weighed and the yield was calculated (**Figure 7B**).

### Fruit diameter and weight measurements

Fruit dimensions were measured from top to bottom (calyx to stigma trace; length) and along the mid-fruit equator (diameter) using a high-precision digital caliper (MRC, MT-110051). Fresh and dry weight of the fruit and seed were measured using a precision balance (LE Series, Sartorius or Boeco, BPS 41 Plus). For dry weight and WC calculation, samples were dehydrated using an air-vacuumed oven (Zenith lab, DZF-6050 or WTC Binder, VD-53) for a minimum of 48 h, at 70°C.

### Total soluble solids measurements

For destructive TSS (Brix units) measurement, fruit pericarp was scraped from top to bottom (from the proximal to the distal side of the fruit). For TSS values <32, measurements were taken using a digital refractometer PR-32α (Atago Co. Ltd.), while for TSS values >32, measurements were taken using an optical refractometer MOD. 103, 0-80 BRIX (Giorgio Bormac S.R.L.).

### Fruit color measurements

Relative fluorescence of detached fruit was measured using a hand-held fluorimeter to estimate chlorophyll levels [Multiplex Research, Force-A; as previously described by (Bahar et al., 2012)]. The chlorophyll index representing fruit exocarp chlorophyll levels is the calculated SFR_G (simple fluorescence ratio – green excitation) value. Spectral color, using the L*, a*, b* color space coordinates, was measured using either an i1 Pro Rev E Chroma meter (x-rite, PANTON^®^ during the 2017 season) or a CR-400 Chroma meter (Konica Minolta, Inc. during the 2018 and 2020 seasons).

### Histology

Whole fruit and pericarp surfaces were examined using a binocular dissection scope (Nikon, SMZ 1270) and imaged with an attached camera (NIKON, DS-Ri2). Samples were stained with the water-soluble Safranin-O dye (Sigma-Aldrich) to examine xylem transport towards the fruit. Six strands were sampled at each time point along the course of fruit development. Three of the strands were placed for 4 h in 5 ml 1% Safranin-O solution and three were placed in 5 ml deionized water as a control. To monitor Safranin-O transport along the xylem and into the fruit pericarp tissue, a longitudinal section of the fruit (through the attached strand segment and at the center of the calyx to the distal end, the stigma trace) and a cross-section (at the middle between the calyx and the distal end) were generated and photographed using zoom stereomicroscope (SMZ1270, Nikon) equipped with a camera (DS-Ri2, Nikon). Fresh lemon juice was applied to avoid oxidation and pericarp browning as a result of the cut.

### ABA quantification

Pericarp tissue (Grofit, 2017 season) was collected at different stages in fruit development (12, 14, 16, 18, 20, 22 and 24 WPP) and frozen in liquid nitrogen. The tissue was ground to a fine powder using a mortar and pestle, after which, 200 mg were weighed and placed in a 2 ml Eppendorf tube containing 1 ml extraction solvent (ES; 80% methanol: 19% water: 1% acetic acid) supplemented with 20 ng of ABA isotope standard (Olchemim). The tube was incubated on Vortex for 60 min at 4°C and the supernatant was collected following centrifugation. The pellet was washed twice with 0.5 ml ES. The collected supernatant was concentrated with a speed-vac concentrator (5301, Eppendorf), at 30°C. The samples were then dissolved in 200 μL solution of 50% methanol. Following centrifugation, the supernatant was filtered through a 0.22 µm PVDF syringe filter and then subjected to LC-MS analysis conducted using a UPLC-Triple Quadrupole-MS (Waters Xevo TQ-MS). Separation was performed on a Waters Acquity UPLC BEH C18 1.7 µm 2.1x100 mm column with a VanGuard precolumn (BEH C18 1.7 µm 2.1 x 5 mm). Chromatographic and MS parameters were as follows: the mobile phase consisted of water (phase A) and acetonitrile (phase B), both containing 0.1% formic acid in gradient elution mode. The flow rate was 0.3 ml min^-1^, and column temperature was 35°C. All analyses were performed using the ESI source in positive ion mode at the following settings: capillary voltage 3.1 KV, cone voltage 30 V, solvation temperature 400°C, solvation gas flow 565 L/h, source temperature 140°C. Quantification was performed using MRM acquisition by monitoring the 247/187, 247/173 (RT=3.92, dwell time of 78 msec for each transition) for ABA, and 253/206, 253/234 (RT=3.92, dwell time of 78 msec) for d6-ABA (used as internal standard). Acquisition of LC-MS data was performed using the MassLynx V4.1 software (Waters).

### Statistical analysis

The Tukey-Kramer HSD test was performed *via* JMP Pro software (Statistical Discovery™, SAS).

## Data availability statement

The raw data supporting the conclusions of this article will be made available by the authors, without undue reservation.

## Author contributions

SE, AB and SH-S planned and designed the research. SE performed the experiments with the assistance of YM, ID, YB, MC and FS, SE, YB, AB and SH-S contributed to the experimental design and data interpretation. SE, AB and SH-S wrote the manuscript. All authors contributed to the article and approved the submitted version.
