# Supplementary material for: Abscisic acid plays a key role in the regulation of date palm fruit ripening
Source: Front Plant Sci. 2023 Feb 17;13:1066142. doi: 10.3389/fpls.2022.1066142 (PMC9981646; doi:10.3389/fpls.2022.1066142)
Supplement: Supplementary file 1 [file Presentation_1.pdf]

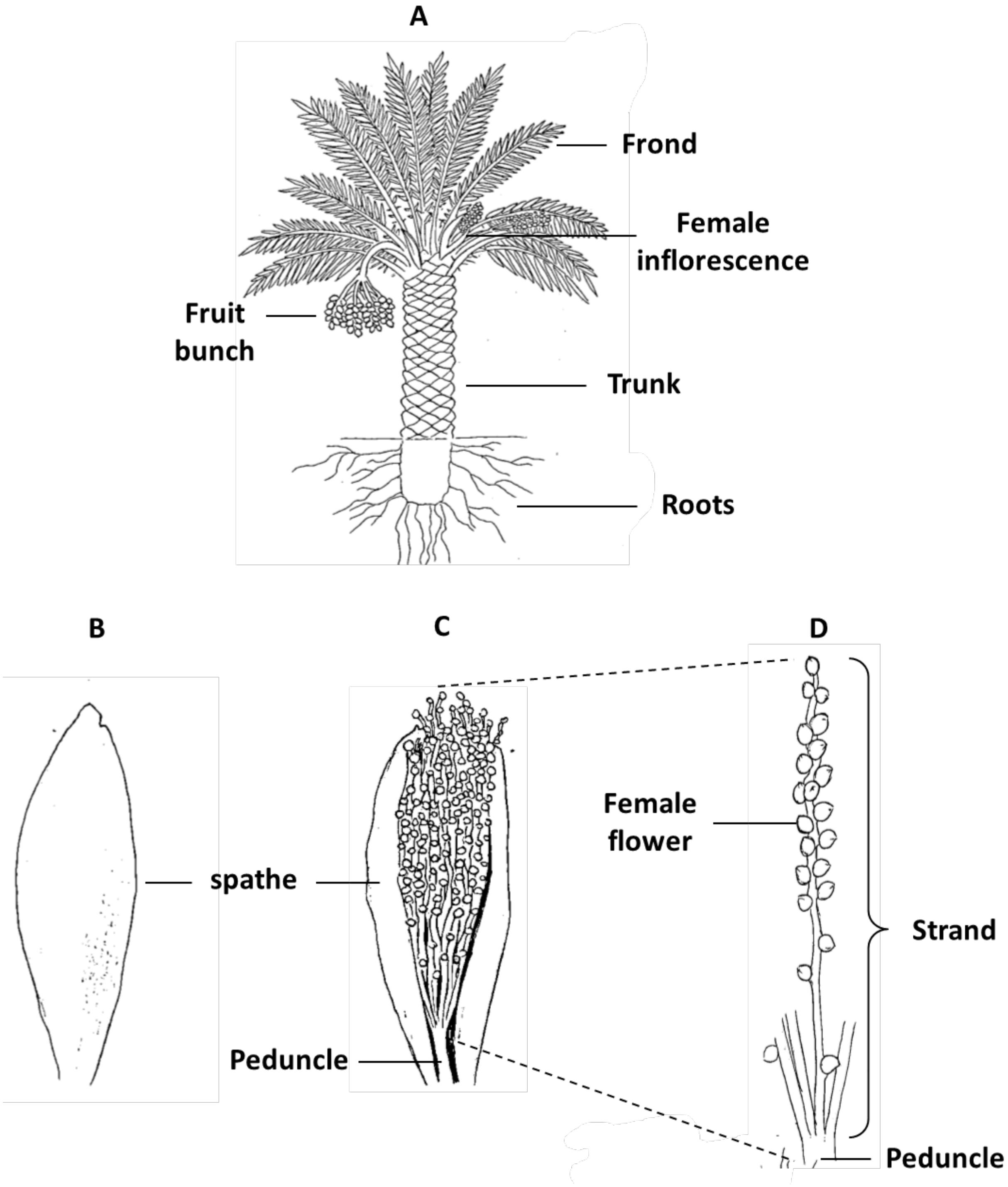

Figure S1. Schematic representation of date palm anatomy and terminology. The date tree **(A)**, closed date spathe **(B)**, open spathe **(C)** and a blowup of single strand with female flowers **(D)**.

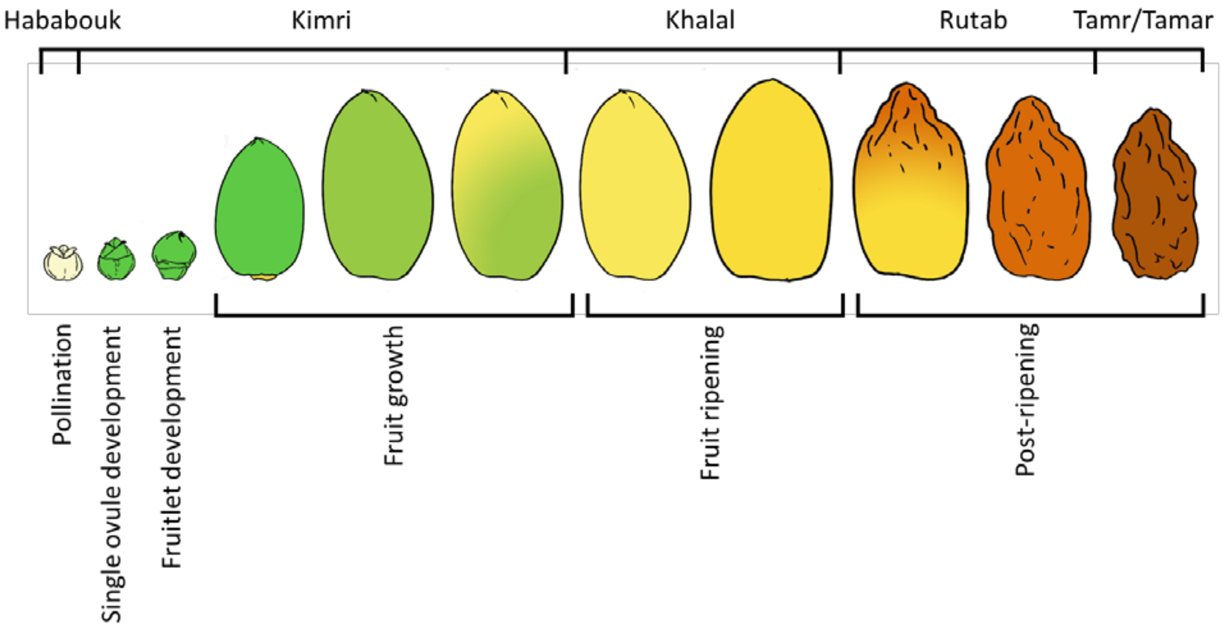

Figure S2. The main stages in date palm fruit development. An illustration of different stages in date fruit development, starting from the female flower at pollination and until the final stages of fruit ripening. Above: the traditional nomenclature for date palm fruit development.

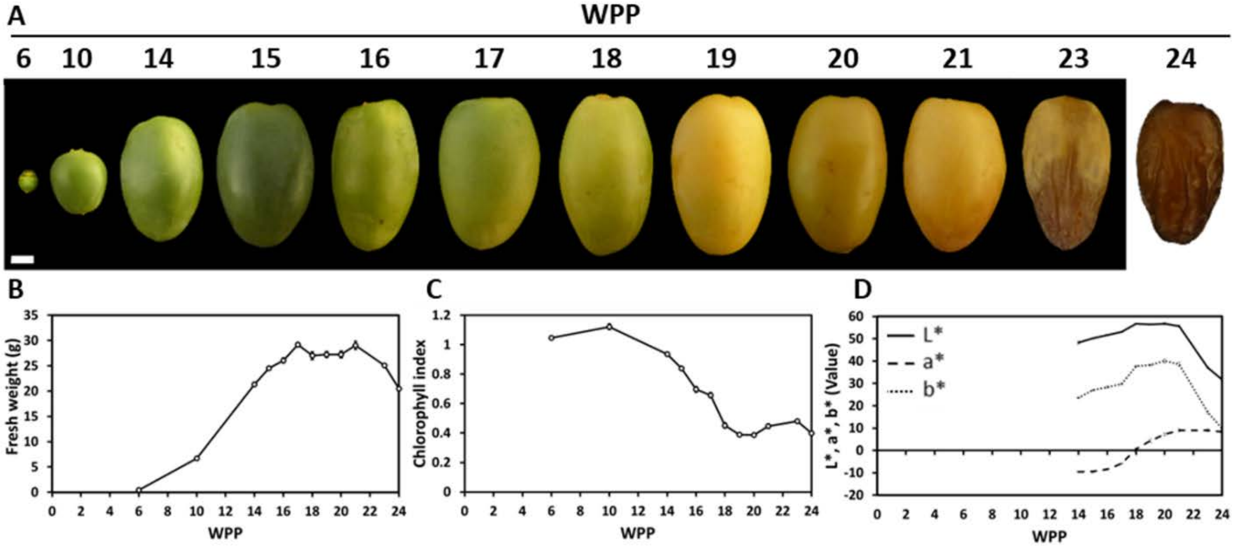

Figure S3. Date palm fruit development during the 2018 season. Representative fruits were sampled and photographed along the course of fruit development, in the Grofit orchard, during the 2018 season. Numbers indicate weeks post pollination **(**WPP; **A)**. Fruit development was characterized through quantification of: fruit fresh weight **(B)**, chlorophyll relative fluorescence **(C)** and the L*a*b* color indices **(D)**. Error bars indicate standard error. Scale bar: 1 cm **(A)**. Number of fruits measured at each time-point is n=30.

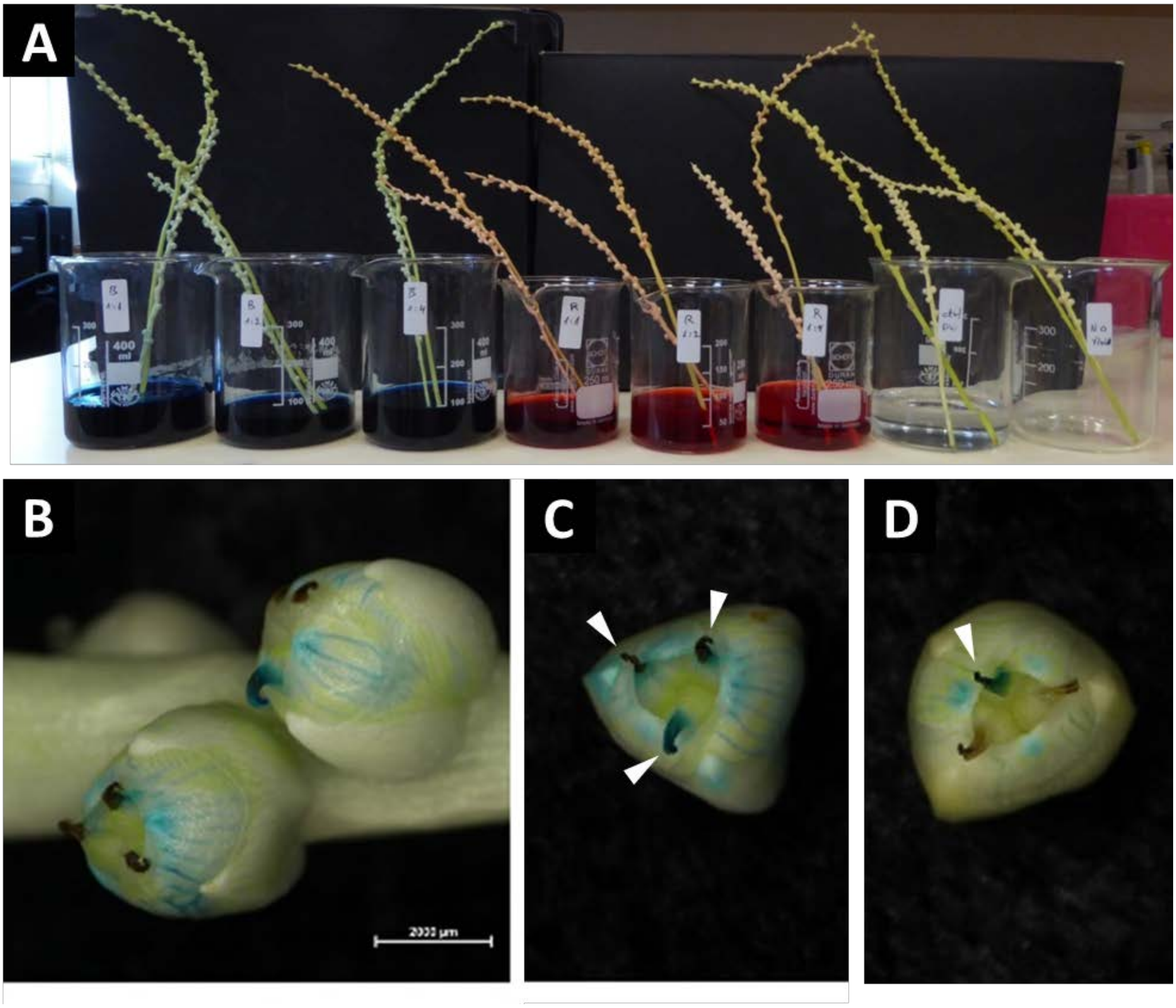

Figure S4. Dye-facilitated assessment of vascular water flux from the strand to the developing fruit. Representative female flowers bearing strands were sampled at pollination (Grofit 2017) and their base was dipped in red or blue water-soluble food dye in order to follow water supply to the developing fruit **(A)**. Flowers were photographed on the strand **(B)** or separated **(C, D)**. Notice the blue dye reaching the calyx scales and either all three stigmas **(C)** or only one of the three **(D)**, all marked by arrows. Scale bar: 0.2 cm **(B, C, D)**.

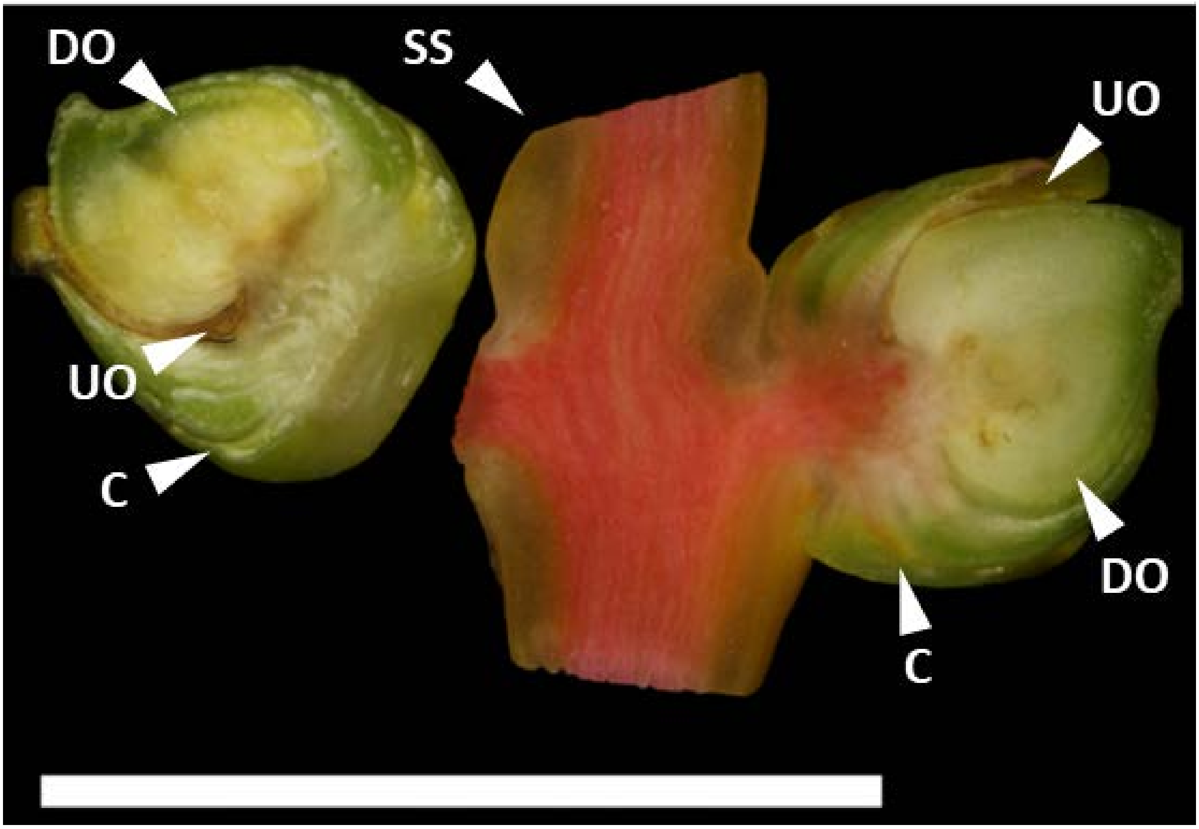

Figure S5. Vascular water flux is disrupted prior to date fruitlet drop. Representative fruitlet bearing strands were sampled at 5 WPP (Grofit, 2017) and water-soluble food dye movement was monitored to follow water supply to the developing fruit. The dye supply to the fruit is shown in a longitudinal section of the fruitlet and its adjacent strand section (SS). Note the differences in dye uptake between the two fruitlets: on the left – a fruitlet that detached in response to a slight touch, while on the right – a persisting fruitlet. Developed ovary - DO, undeveloped ovary - UO, calyx - C. Scale bar: 1 cm.

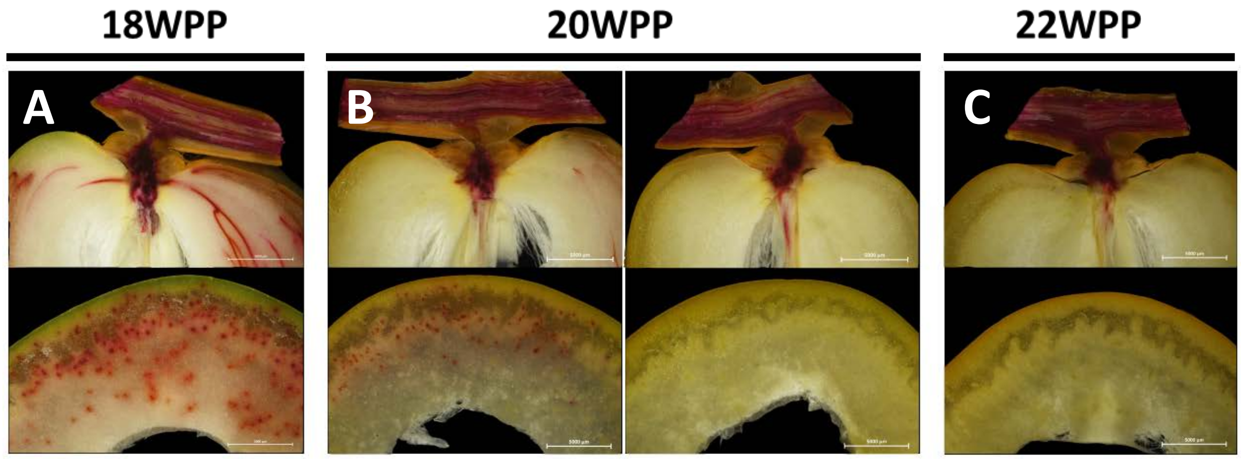

Figure S6. Vascular water flux arrest from the strand to the developing date fruit. Representative fruit bearing strands were sampled along the course of fruit development (Grofit, 2018) and movement of the dye Safranin O was monitored at different stages in fruit development using cross and longitudinal sections. Cross-sections of fruits sampled at 18 **(B)**, 20 **(C)** and 22 **(D)** weeks post pollination (WPP) are presented. Scale bar: 0.5 cm. Number of sampled strands at each time-point was n=3, each strand bearing 6-15 date fruits.

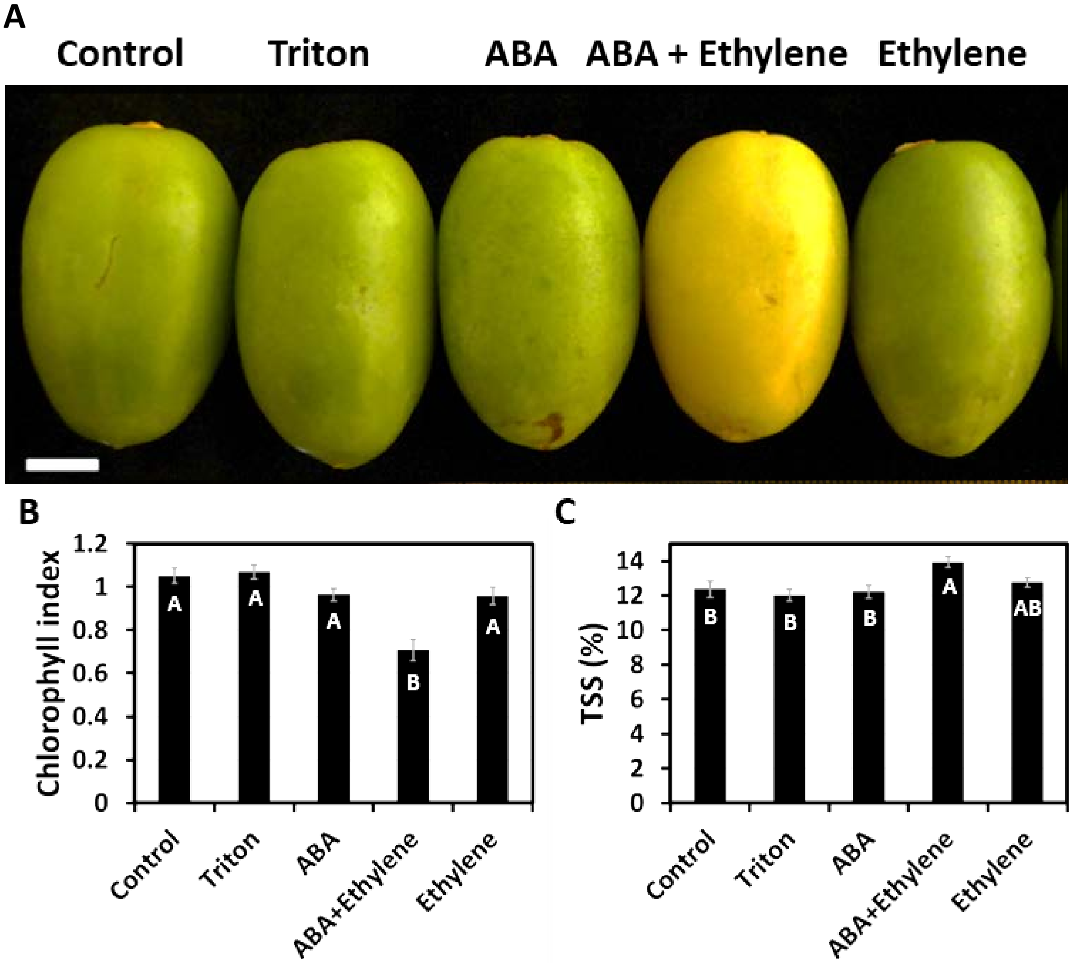

D

Treatment	L*		a*		b*	
Control	52.74 B	±0.41	-12.83 B	±0.30	14.74	±0.60
Triton	54.69 AB	±0.62	-11.43 AB	±0.34	13.60	±0.65
ABA	54.45 AB	±0.71	-11.24 AB	±0.35	15.10	±0.72
ABA + Ethylene	55.87 A	±0.51	-10.11 A	±0.44	16.12	±1.11
Ethylene	54.08 AB	±0.82	-11.64 AB	±0.41	14.41	±0.59

Figure S7. The effect of exogenous ABA and ethylene treatment applied 14 WPP on date fruit development. Date fruits were treated with ABA (ProTone^{TM}), ethylene (Ethrel®) and their combination, at 14 WPP, on the tree (Grofit, 2017). Representative fruits were sampled and photographed one week after application **(A)**. Effects were characterized through quantification of: chlorophyll relative fluorescence **(B)**; total soluble solids (TSS; %; **C)**; color index **(D)**. TSS scores are equivalent to Brix units as measured by a refractometer. Error bars and "±" indicate standard error. Letters represent Tukey-Kramer multiple comparison test **(B**; p-value $\leq$ 0.0001, **C**, **D**; p-value $\leq$ 0.05**)**. Scale bar: 1 cm **(A)**. Number of fruits measured per treatment at each time-point was n=12 **(B, D)** and n=6 **(C)**.

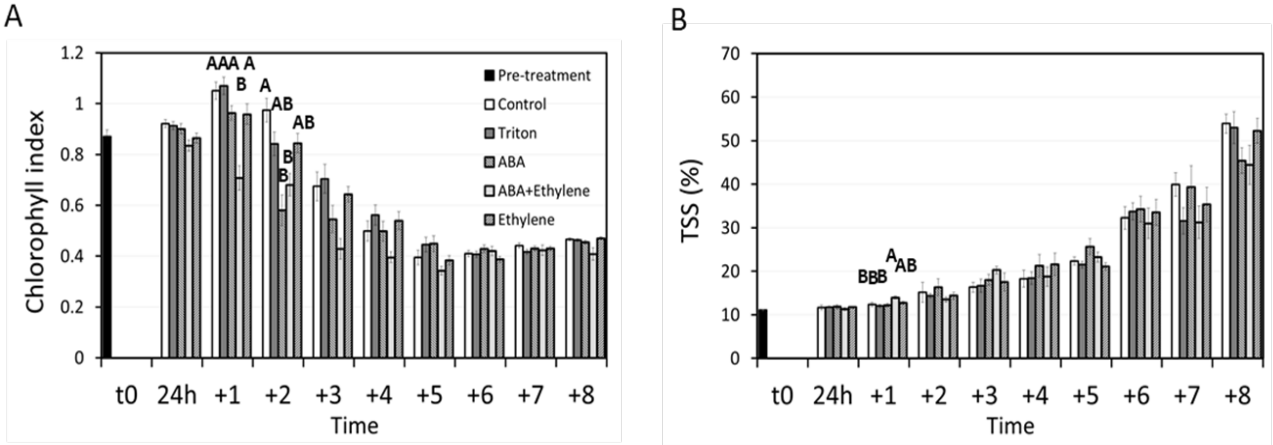

C

Treatment	a*																			
	t0		24h		+1		+2		+3		+4		+5		+6		+7		+8	
Pre-treatment	-15.07	±0.19																		
Control			-14.79	±0.42	-12.83 B	±0.30	-15.41 B	±0.18	-13.28 B	±0.31	-10.50 AB	±0.49	-7.46	±0.29	-5.89	±0.24	-4.55	±0.23	-3.95	±0.25
Triton			-14.21	±0.41	-11.43 AB	±0.34	-14.46 B	±0.50	-12.80 B	±0.35	-10.91 B	±0.61	-8.13	±0.49	-4.91	±0.34	-4.38	±0.26	-3.48	±0.33
ABA			-14.24	±0.25	-11.46 AB	±0.28	-12.38 A	±0.50	-11.74 AB	±0.44	-10.14 AB	±0.59	-8.08	±0.63	-4.46	±0.44	-4.17	±0.32	-3.81	±0.30
ABA + Ethylene			-14.72	±0.22	-10.43 A	±0.31	-12.85 A	±0.31	-9.76 A	±0.58	-7.40 A	±0.34	-6.08	±0.67	-5.44	±0.31	-3.15	±0.50	-3.80	±0.26
Ethylene			-14.15	±0.28	-11.64 AB	±0.41	-14.77 B	±0.21	-12.52 A	±0.29	-10.98 B	±0.38	-7.25	±0.41	-5.51	±0.29	-4.24	±0.26	-3.69	±0.31

Treatment	b*																			
	t0		24h		+1		+2		+3		+4		+5		+6		+7		+8	
Pre-treatment	15.63	±0.48																		
Control			15.23	±0.59	14.74	±0.60	18.01 B	±0.27	21.03 B	±0.43	22.09 AB	±0.83	25.61	±0.35	25.04 B	±0.29	26.17	±0.36	24.58	±0.33
Triton			15.19	±0.67	13.60	±0.65	18.96 B	±0.49	21.34 AB	±0.51	23.53 B	±0.49	25.14	±0.48	25.57 AB	±0.35	26.90	±0.36	21.56	±1.62
ABA			15.35	±0.43	15.10	±0.72	22.07 B	±0.44	22.92 AB	±0.44	24.42 B	±0.44	25.08	±0.42	25.90 AB	±0.38	27.21	±0.37	25.11	±1.06
ABA + Ethylene			17.06	±0.56	16.12	±1.11	21.70 A	±0.40	24.12 A	±0.55	18.08 A	±1.11	26.19	±0.59	26.99 A	±0.42	27.11	±0.43	25.66	±0.45
Ethylene			15.95	±0.34	14.41	±0.59	19.48 A	±0.38	21.58 AB	±0.37	24.29 B	±0.40	25.88	±0.37	25.89 AB	±0.31	26.30	±0.35	24.11	±0.67

Figure S8. The continuous affect of exogenous hormonal treatments applied 14 WPP on date fruit ripening. Date fruits were treated on the tree with ABA (ProTone^{TM}), ethylene (Ethrel®) and their combination, at 14 WPP (Grofit 2017). Representative fruits per treatment were sampled before treatment (t0), after 24 hours (24h) and every week (+1, +2…) until harvesting time. Fruit ripening was characterized through quantification of: chlorophyll relative fluorescence **(A)**; total soluble solids **(**TSS; %; **B)**; and, color index **(C)**. TSS scores are equivalent to Brix units as measured by a refractometer. Error bars and "±" indicate standard error. Letters represent Tukey-Kramer multiple comparison test; p-value $\leq$ 0.05 . Number of fruits measured per treatment at each time-point was n=12 **(A, C)** and n=6 **(B)**.

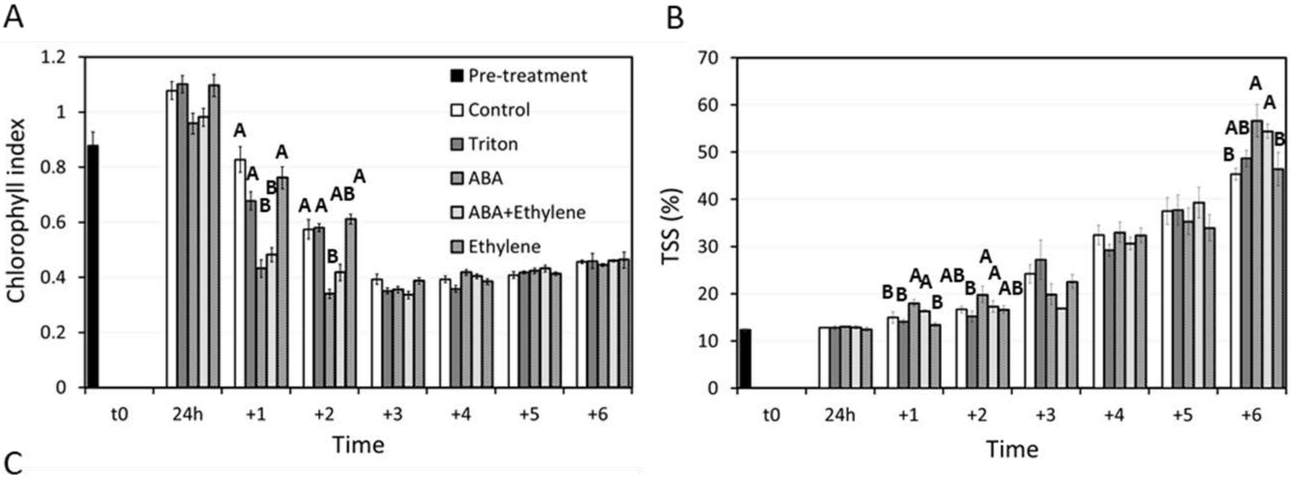

C

Treatment	a*															
	t0		24h		+1		+2		+3		+4		+5		+6	
Pre-treatment	-15.47	±0.20														
Control			-16.14	±0.21	-13.81 B	±0.32	-12.33 B	±0.32	-9.32 B	±0.37	-6.32	±0.26	-5.29	±0.22	-5.06	±0.19
Triton			-15.95	±0.17	-13.71 B	±0.22	-10.22 A	±0.41	-8.55 AB	±0.32	-6.18	±0.27	-5.14	±0.21	-4.50	±0.23
ABA			-15.64	±0.17	-11.60 A	±0.32	-9.59 A	±0.39	-7.30 A	±0.40	-6.05	±0.25	-5.71	±0.22	-5.29	±0.27
ABA + Ethylene			-15.60	±0.17	-11.80 A	±0.33	-11.93 B	±0.38	-7.90 AB	±0.31	-5.24	±0.29	-5.25	±0.21	-4.59	±0.23
Ethylene			-16.01	±0.16	-13.62 B	±0.29	-12.09 B	±0.23	-9.14 B	±0.37	-5.44	±0.28	-5.15	±0.24	-4.26	±0.27

Treatment	b*															
	t0		24h		+1		+2		+3		+4		+5		+6	
Pre-treatment	17.83	±0.28														
Control			16.31	±0.26	18.84 B	±0.46	22.28 B	±0.41	24.57	±0.48	25.42	±0.25	25.65	±0.30	24.89	±0.21
Triton			16.60	±0.29	19.67 B	±0.45	23.68 AB	±0.33	25.02	±0.43	26.01	±0.28	26.45	±0.21	24.69	±0.37
ABA			17.30	±0.24	22.28 A	±0.46	24.19 A	±0.32	25.27	±0.28	25.20	±0.30	26.24	±0.22	23.77	±0.54
ABA + Ethylene			17.45	±0.26	21.91 A	±0.36	22.84 AB	±0.45	25.83	±0.32	26.32	±0.33	26.33	±0.31	24.43	±0.28
Ethylene			16.91	±0.22	19.06 B	±0.31	22.14 B	±0.37	24.74	±0.46	25.80	±0.30	26.22	±0.23	25.42	±0.40

Figure S9. The continuous effect of exogenous hormonal treatments applied 16 WPP on date fruit ripening. Date fruits were treated on the tree with ABA (ProTone™), ethylene (Ethrel®) and their combination, at 16 WPP (Grofit 2017; see figure 5). Representative fruits per treatment were sampled before treatment (t0), after 24 hours (24h) and every week (+1, +2…) until harvesting time. Fruit ripening was characterized through quantification of: chlorophyll relative fluorescence **(A)**; total soluble solids **(**TSS; %; **B)**; and, color index **(C)**. TSS scores are equivalent to Brix units as measured by a refractometer. Error bars and "±" indicate standard error. Letters represent Tukey-Kramer multiple comparison test; p-value $\leq$ 0.05. Number of fruits measured per treatment at each time-point was n=12 **(A, C)** and n=6 **(B)**.

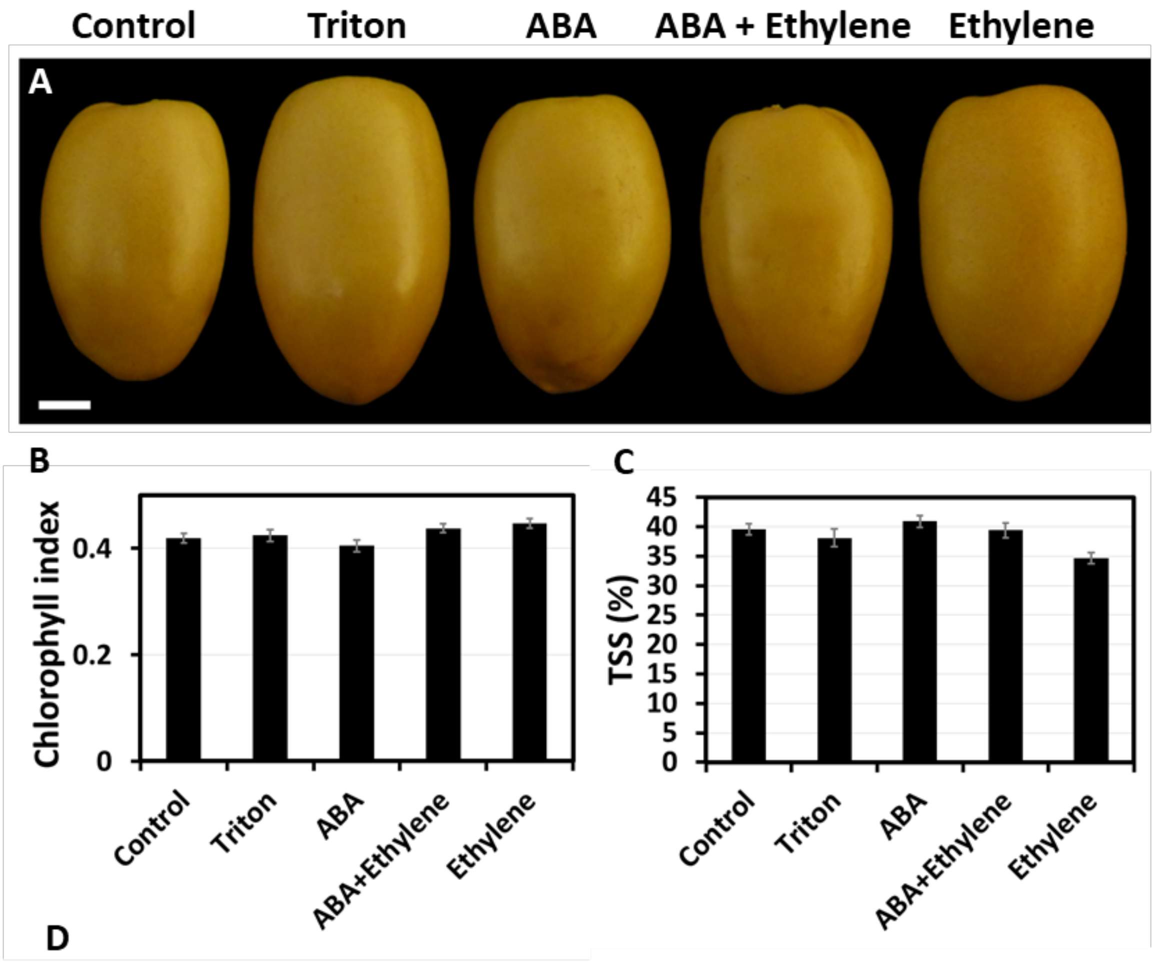

D

Treatment	L*		a*		b*	
Control	54.58	±0.19	-5.19	±0.33	24.30	±0.36
Triton	54.50	±0.21	-5.41	±0.18	25.52	±0.32
ABA	54.29	±0.21	-5.16	±0.20	24.77	±0.34
ABA + Ethylene	54.64	±0.18	-5.30	±0.25	24.88	±0.34
Ethylene	54.71	±0.20	-4.97	±0.27	25.06	±0.25

Figure S10. The effect of exogenous ABA and Ethylene treatment applied 20 WPP on date fruit development. Date fruits were treated with ABA (ProTone^{TM}), ethylene (Ethrel®) and their combination, at 20 WPP on the tree (Grofit 2017). Representative fruits per treatment were sampled and photographed one week after the treatment **(A)**. Fruit ripening was characterized through quantification of: chlorophyll relative fluorescence **(B)**; total soluble solids (TSS; %; **C)**; and, color index **(D)**. TSS scores are equivalent to Brix units as measured by a refractometer. Error bars and "±" indicate standard error. Scale bar: 1 cm **(A)**. Number of fruits measured per treatment at each time-point was n=12 **(B, D)** and n=6 **(C)**.

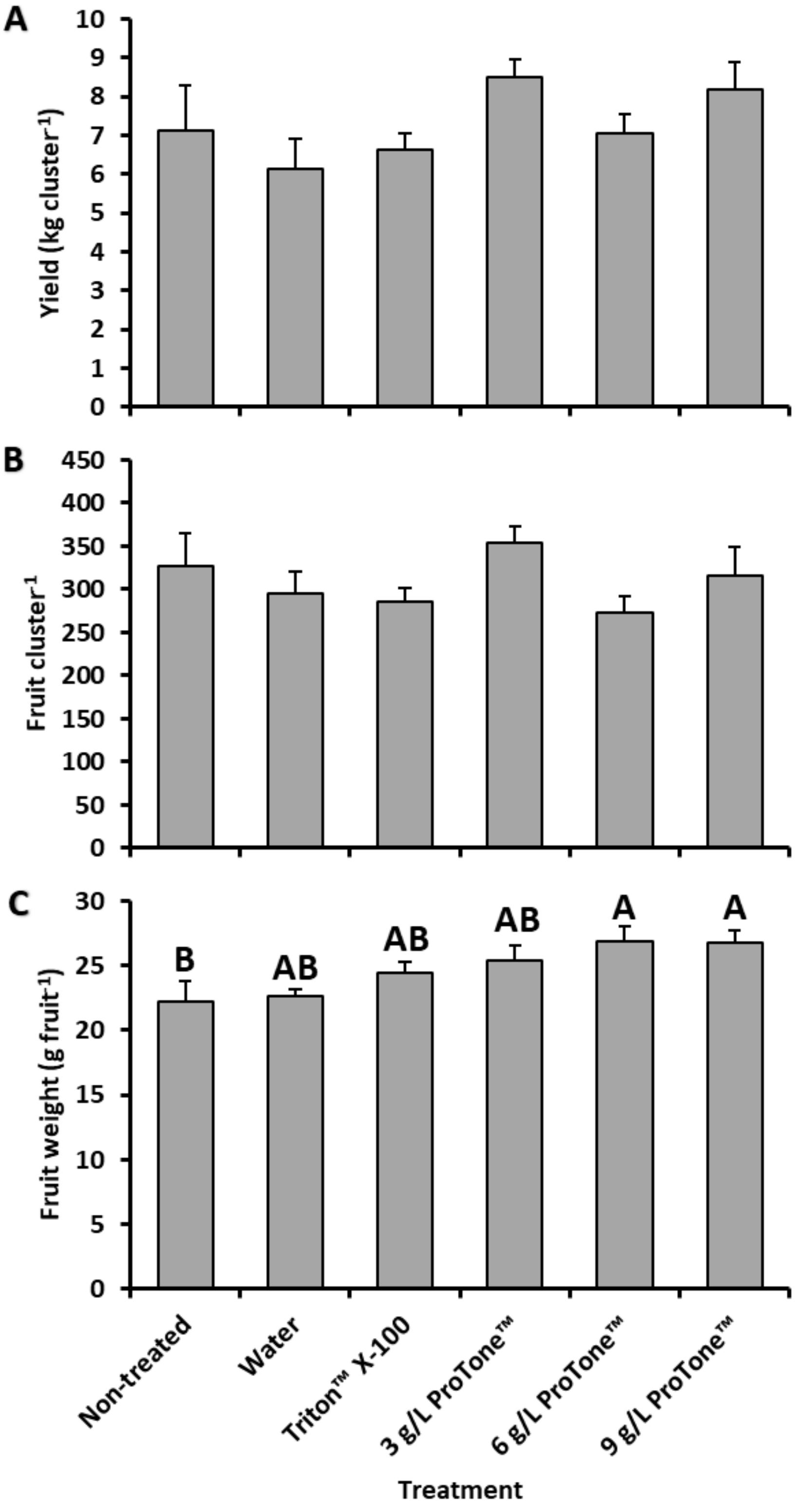

Figure S11. **The effect of pre-harvest ABA treatments on major yield parameters.** In the season of 2020, whole fruit clusters were treated pre-harvest every three days (starting from 17 WPP, four rounds in total) with ABA (ProTone™; 3, 6 and 9 g/L) and 0.1% Triton X-100. The treated fruits were compared to the corresponding controls: 0.1% Triton™ X-100, water and non-treated fruit. Fruit yield **(A)**; number of fruit per cluster **(B)**; and, mean fruit fresh weight **(C)** are presented. Letters represent Tukey-Kramer multiple comparison test (**C**; p-value $\leq$ 0.05**)**. Values are means of 10 replicates (clusters). Bars represent standard error.

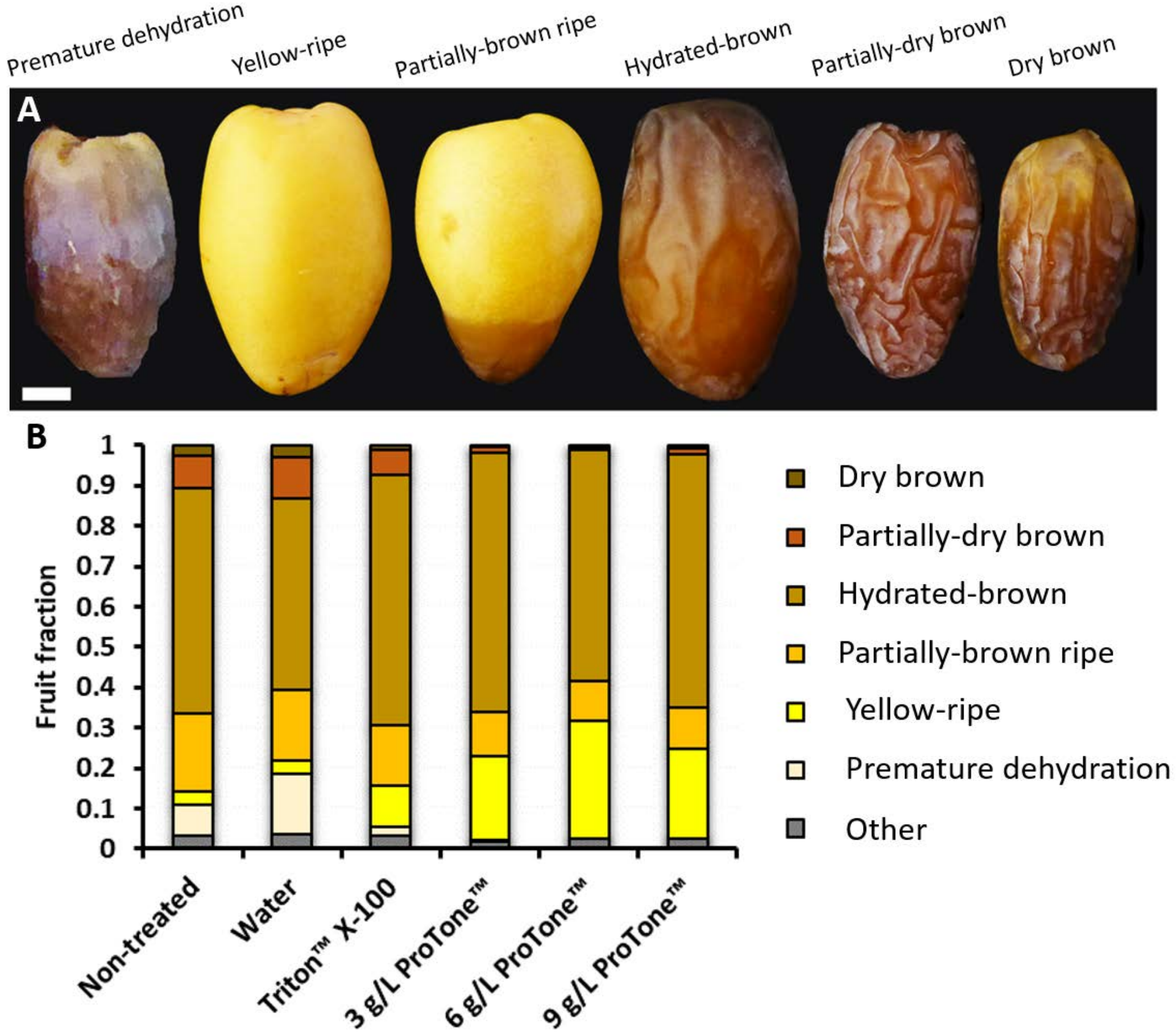

Figure S12. **The effect of ABA treatment on fruit ripening at harvest.** In the season of 2020, whole fruit clusters were treated pre-harvest every three days (starting from 17 WPP, four rounds in total). On the second harvest round (out of five in total), the fruit was sorted into seven classes by appearance and texture **(A)** and for each treatment the fraction of each of the seven classes was calculated **(B)**. Treatments included: non-treated control; water; Triton™ X-100 (0.1%); and ProTone™ at 3; 6; and 9 g L^{-1}, with Triton™ X-100 (0.1%). The number of clusters harvested for each treatment is n=10.
